# Repetitive Bouts of Exhaustive Exercise Induces a Systemic Inflammatory Response and Multi-Organ Damage in Rats

**DOI:** 10.3389/fphys.2020.00685

**Published:** 2020-06-23

**Authors:** Peng Liao, Qinghua He, Xuan Zhou, Kai Ma, Jie Wen, Hang Chen, Qingwen Li, Di Qin, Hui Wang

**Affiliations:** ^1^Research Center for Sports Nutrition and Eudainomics, Institute for Sports Training Science, Tianjin University of Sport, Tianjin, China; ^2^Jiangsu Biodep Biotechnology, Jiangyin, China; ^3^Probiotics Australia, Ormeau, QLD, Australia; ^4^Beijing Allwegene Health, B-607 Wanlin Technology Mansion, Beijing, China; ^5^Beijing Tong Ren Tang Health-Pharmaceutical, Beijing, China; ^6^Department of Pharmacology, School of Pharmacy, Nantong University, Nantong, China

**Keywords:** exhaustive exercise, systemic inflammation, multi-organ dysfunction, necrosis, microhemorrhage

## Abstract

Multiple organ dysfunction syndrome can follow severe infection or injury, but its relationship to exercise is not well understood. Previous studies have observed that prolonged strenuous exercise can lead to transiently increased level and/or activity of markers for systemic inflammatory response and multiple organ damage. However, few studies have analyzed the pathogenesis of the inflammatory response and subsequent multi-organ injury in exhaustive exercise conditions. In this study, we established a rat model of repetitive bouts of exhaustive running (RBER) and investigated its effects on multiple organ damage. Rats were subjected to RBER in either uphill or downhill running modes daily for a period of 7 days. Morphologically, RBER causes tissue structural destruction and infiltration of inflammatory cells in the skeletal muscles and many visceral organs. RBER also causes sustained quantitative changes in leukocytes, erythrocytes, and platelets, and changes in the concentration of blood inflammatory factors. These inflammatory alterations are accompanied by increases in serum enzyme levels/activities which serve as functional markers of organ damage. In general, RBER in the downhill mode seemed to cause more damage evaluated by the above-mentioned measures than that produced in the uphill mode. A period of rest could recover some degree of damage, especially for organs such as the heart and kidneys with strong compensatory capacities. Together, our data suggest that, as a result of multi-organ interactions, RBER could cause a sustained inflammatory response for at least 24 h, resulting in tissue lesion and ultimately multiple organ dysfunction.

## Introduction

Exercise is highly regarded as a lifestyle modification which can significantly benefit one’s health; lack of exercise has been shown to be a contributing factor in a variety of disease processes including metabolic syndrome, type 2 diabetes mellitus, heart disease/failure, vascular disease, and cognitive decline (Booth et al., 2012). Adoption of an exercise regimen is often an early strategy for reversing or preventing disease progression. This is perhaps most well documented in the setting of cardiovascular disease, where enhanced physical activity has been shown to be effective in primary prevention of coronary artery disease (CAD) as well as in the secondary reduction in progression and impact of the disease (Alves et al., 2016). These beneficial effects that arise from exercise are the result of its ability to stimulate the internal environment and induce widespread, integrated changes in metabolism, cardiovascular tone, and pulmonary function.

However, excessive exercise which exhausts the body’s adaptive reserve, or ability to respond positively to a training stimulus, can be damaging. While this adaptive reserve is relatively high in healthy, young adults, some conditions such as fatigue, disease states, acute or chronic exhaustive training, and lack of adequate recovery following exercise can compromise the adaptive reserve and predispose individuals to exercise-induced organ dysfunction and injury (Marques et al., 2018). An objective definition of exhaustive exercise is difficult as it is, in humans, measured volitionally by the subjects’ inability to maintain the exercise regimen. This is typically in association with quantification of the subjects’ maximal oxygen consumption, or VO_2_ max, which is a measurement of the amount of oxygen a person can utilize during intense exercise (Stawski et al., 2017). In animal models, exhaustive exercise has typically been evaluated by treadmill running or forced swimming exercises. In these paradigms, animals are said to be exhausted when they refuse to continue running, lack coordinated movements, or sink to the bottom of the pool (Huang et al., 2013; Yan and Hao, 2016; Xia et al., 2018). The harmful effects of exhaustive exercise are pervasive across a variety of body systems, including musculoskeletal, cardiovascular, pulmonary, hematologic, metabolic, and gastrointestinal. Skeletal muscle damage resulting from exhaustive exercise is primarily induced by mechanical stretch injury. Such damaging exercise results in direct injury to the sarcomere as well as plasma membrane damage resulting in the release of muscle enzymes, such as creatine kinase (CK), into the plasma (Behringer et al., 2014; Owens et al., 2019). Furthermore, skeletal muscle damage induces an inflammatory response to the muscle necrosis that results in activation and migration of immune cells. While this response is important for muscle regeneration following normal exercise, excessively damaging or exhaustive exercise induces a larger, systemic inflammatory response with release of a variety of cytokines and chemokines which can induce multi-organ effects (Yang and Hu, 2018). As such, beyond the direct damage to skeletal muscle, exhaustive exercise is known to induce systemic phenomena such as heat stress (American College of Sports Medicine et al., 2007; Heytens et al., 2017), visceral organ ischemia via splanchnic hypoperfusion (van Wijck et al., 2012), and alterations in blood cell counts and inflammatory mediator levels (Gannon et al., 1997; Weinstock et al., 1997; Ronsen et al., 2001).

The blood and its immune components function as a multi-organ network linking the various bodily systems. Functions include delivery of oxygen and nutrients to tissues, transport of waste products for excretion, and of course, response to pathogenic or injurious stimuli via circulating immune cells and mediators. In the acute setting of injury or infection, local tissue-based and circulatory innate immune cells are rapidly activated in the presence of stressful stimuli, and trigger a larger systemic inflammatory response in the initial phase to remove the injury stimulant, protect tissue, repair damage, and restore homeostasis (Eppensteiner et al., 2018). However, with extensive tissue injury as occurs in severe trauma or systemic insults, excessive activation of the inflammatory response leads to prolonged immune cell activation and release of various pro- and anti-inflammatory cytokines and chemokines. Such widespread inflammation, typically resulting from the release of endogenous damage-associated molecular patterns (DAMPs) that activates both immune cells (i.e., neutrophils and monocytes) and complement, can exacerbate damage to the injured tissue while also invoking damage in other organs. This phenomenon is referred to as the systemic immune response syndrome (SIRS), and if persistent, can progress to multiple organ dysfunction (MOD) and death (Lord et al., 2014).

Exercise-induced muscle damage and inflammation is a well-documented phenomenon, with reports of post-exercise leukocytosis dating back to 1893. In addition to leukocytosis, acute bouts of exercise have been shown to increase levels of inflammatory mediators substances such as C-reactive protein (CRP) and interleukins 1 and 6 (IL-1, 6) (Malm, 2001). Further studies have identified increased circulating levels of cell free DNA, a potent DAMP, following acute physical activity, with DNA being derived from both hematopoietic lineage cells and mitochondria (Tug et al., 2015; Shockett et al., 2016). This pro-inflammatory effect of exercise is considered to be transient though, and several studies have shown that regular exercise results in a net reduction in pro-inflammatory markers while enhancing the level of anti-inflammatory substances such as IL-10 and adiponectin. It is hypothesized that this anti-inflammatory response is necessary for limiting muscular damage and promoting muscle recovery and regeneration (Ploeger et al., 2009; Sallam and Laher, 2016). In cases of persistent/repetitive exhaustive exercise or overtraining, where the recovery period may be insufficient, the inflammatory response may promote harmful systemic inflammation with the potential for SIRS and MOD, while leaving individuals prone to infection due to a persistent immunocompromised state.

While exercise is known to induce inflammation and such inflammation may eventually lead to multi-organ damage, few studies have analyzed the pathogenesis of this condition (Behringer et al., 2016; Shockett et al., 2016; Couto et al., 2018; Goh and Behringer, 2018). Important questions about the role of various types of exercise, exercise load or intensity, and lack of recovery periods in transient inflammation and the potential link to organ injury and MOD remain. Specifically, the ability of exhaustive exercise to induce sustained changes in inflammation and subsequent alterations consistent with multi-organ injury has not been explored. To contribute a new layer of knowledge to this topic, here we have exposed rats to repetitive sessions of exhaustive exercise in order to induce a systemic inflammatory response. We hypothesized that the repetitive bouts of exhaustive exercise, occurring at intervals of 24 h via uphill and downhill running, would prohibit adequate post-exercise recovery, and lead to multi-organ injury consistent with the clinical syndrome of MOD. Using this model, we hereby demonstrate evidence for the induction of systemic inflammation by repetitive exhaustive exercise which is associated with system-wide injuries to various organs.

## Materials and Methods

### Animal Care and Grouping

**FIGURE 1:**
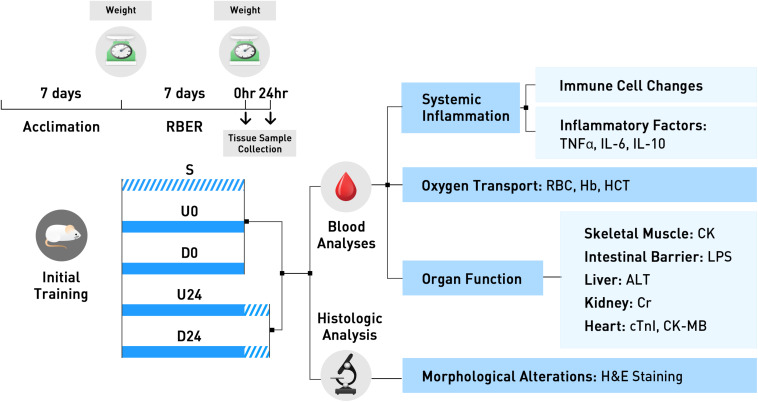
The experimental design and conduct of this study is exhibited here. All animals were trained for 7 days during the acclimation period. Animals were then randomly assigned to the non-exercise sedentary group, or to 1 of 4 exercise groups. Animals in U0 and D0 were trained using the repetitive bouts of exhaustive running (RBER) model for 7 days, via uphill and downhill running respectively, and sacrificed immediately after. Animals in the U24 and D24 were similarly trained, but were sacrificed after 24 h of rest. Tissues were collected following sacrifice and processed for various blood and histologic analyses. S, sedentary; U0, uphill training, samples collected immediately; U24, uphill, 24 h later; D0, downhill, immediately; D24, downhill, 24 h later.

The protocol for this study was approved by the Committee for the Care and Use of Laboratory Animals at Tianjin University of Sport. Animals were housed in standard cages and maintained on a controlled schedule for light exposure (12-hour light-dark cycles). Animals were fed a diet of distilled water and food (crude protein- no less than 22%, crude fat- no less than 4%, crude fiber- no more than 5%, moisture- no more than 10%, ash- no more than 8%) *ad libitum*. Sixty-four 10-week-old healthy male Sprague Dawley (SD) rats (average weight: 282 ± 25 g) were trained to run on an electric treadmill at a rate of 15 m/min for 15–30 min each day for a week prior to random assignment into one of five groups: the non-exercise sedentary group (S group, *n* = 10), the group examined immediately after uphill running (U0 group, *n* = 13), the group examined 24 h after uphill running (U24 group, *n* = 14), the group examined immediately after downhill running (D0 group, *n* = 14), and the group examined 24 h after downhill running (D24 group, *n* = 13). This animal model and strain is commonly used in exhaustive exercise models. Ten-week animals were used as this represents the age of sexual maturity, and these animals also have optimal aerobic running endurance. The experimental design and conduct of this study is displayed in Figure 1.

### Exercise Modes and Methods

For a period of 7 days following the initial training (described above), the rats were subjected to repetitive bouts of exhaustive running (RBER) via a daily regimen of uphill (+ 10% slope) or downhill (−10% slope) running on a treadmill. Each session began with the animals running at an initial speed of 12 m/min, increasing at a rate of 3 m/min every 5 min to a maximum speed of 27 m/min. The animals were continuously exercised in this way until they met the criteria for exhaustion: decrease in all coordinated movements and/or inability of the animal to stand on its own. At exhaustion, they were permitted to rest for 2–3 min at which point the above described procedure was repeated three more times for a total of four sessions. The animals were exercised in this fashion for 7 days. This model accounts for objective signs of exhaustion to define the duration of training, rather than using a defined time point. Animals weights were calculated prior to engaging in this exercise regimen on Day 0 and Day 7 (at approximately 12:00 pm). The time to exhaustive exercise (i.e., latency between beginning exercise and exhaustion) was calculated during the first (Day 0) and last (Day 7) sessions. These assessments occurred at approximately the same time of day (1:00–4:00 pm) to limit potential influence of Circadian changes.

### Tissue Sample Collection, Preparation and Staining

Animals were anesthetized by ketamine (75 mg/kg) and xylazine (10 mg/kg) and euthanized by exsanguination immediately (groups S, U0, and D0) or 24 h (groups U24 and D24) following completion of the above described 7-day period of exercise. Tissue samples were collected to examine the morphologic, physiological and biochemical changes. Whole blood samples were collected from the abdominal aorta in heparin coated tubes for immediate testing. The remaining samples were fractioned into plasma, erythrocytes, leukocytes, and platelets and stored at −80°C. The right side or part of the kidney (cortex), intestines (1 cm section located 15 cm distal to pylorus), lung (middle lobe), liver (right lobe), heart (cross section midway through), triceps brachii muscle (medial head) and lateral femoris muscle (junction of deep lower tendon and muscle) were collected, rinsed with chilled PBS, and fixed by immersion in prechilled 10% formaldehyde (refreshed after 1 h) overnight while the left side or part of the same structures were immediately frozen in liquid nitrogen and stored at −80°C. Similar regions were isolated from each organ. The fixed tissue was flushed with water, then trimmed, dehydrated, waxed and embedded in paraffin. The tissues were then sectioned at a thickness of 4 μm, classically stained with hematoxylin and eosin (H&E), and evaluated by two independent pathologists in a double-blind approach (i.e., sections were observed with no knowledge of group allocation and the histopathologic changes were confirmed by two independent pathologists) to minimize bias. For histological analysis, 6 slides were randomly selected, slides were surveyed for scatter damage, and 5 visual fields were imaged per slide to minimize bias.

### Blood Cell Analysis, Enzyme Activity, and ELISA

Whole blood samples (20 μL) were hemodiluted with diluent NK (2 mL; Nihon Kohden Co., 8-522) and then measured using the MEK-6318K Automatic Blood Cell Analyzer (Nihon Kohden Co.). The activity/levels of serum creatine kinase (CK), creatinine (Cr), and alanine aminotransferase (ALT) were evaluated with the Beckman Au2700 Automatic Biochemical Analyzer (Beckman Coulter, Inc.) using CK assay kit (Nanjing Jiancheng Biotech. A032), Cr assay kit (Nanjing Jiancheng Biotech. C011-1), ALT assay kit (BioSino Bio-technology. 142331) The serum concentration of tumor necrosis factor alpha (TNF-α; 20140403AB5), lipopolysaccharide (LPS), interleukin 6 (IL-6; 20140421BG1), interleukin 10 (IL-10; 20140423MC1), CK-myocardial band (CK-MB; 20140421ND5), and cardiac troponin I (cTnI; 20140421MG3) was detected via the ELISA kits from Shanghai Zhuokang Biological Technology.

### Statistical Analysis

All quantitative experimental data were processed using the IBM SPSS Statistics Software (SPSS 19.0 for Windows). Descriptive statistics were calculated and include the mean value and standard deviation and are presented in the results as mean ± standard deviation. Differences among all five groups were tested by one-way ANOVA. Paired *t*-tests were followed to evaluate the difference within groups (i.e., for each pair of change in body weight and exercise time). Independent *t*-tests were used to evaluate the difference between two groups. For multiple tests, *p* values were adjusted with the Bonferroni correction. Adjusted *p* values < 0.05 were considered to be statistically significant.

## Results

### Reduction in Body Weight Is Accompanied by Decreased Time to Exhaustion

**FIGURE 2:**
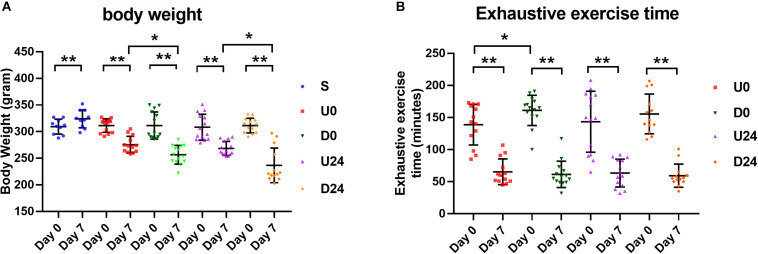
Changes in body weight and exhaustive time in different groups. **(A)** Except the sedentary group (S), the average body weight of rats in each exercise group was decreased significantly after RBER. **(B)** Exhaustive exercise time in each exercise group was significantly reduced at day 7 after RBER. Data is represented as mean ± SD in the figures. U0, uphill training, analyzed immediately; U24, uphill, 24 h later; D0, downhill, immediately; D24, downhill, 24 h later. *P < 0.05; **P < 0.01; *n* ≥ 10.

We first investigated whether the time to exhaustion during exercise was correlated with body weight in our design. Weights were evaluated at the beginning, rats in all groups had a similar average body weight. While rats in the sedentary group S gained weight in 7 days, rats in each exercise group lost between 11.6 and 23.8% of their body weight in the same period (*p* < 0.01; Figure 2A). Notably, the degree of weight loss was significantly greater in the downhill running groups (D0 vs. U0, D24 vs. U24; *p* < 0.01; Figure 2A). As measured on the first day of exercise, the rats in the downhill running groups had a longer exhaustive exercise time on average than those in the uphill running groups, suggesting a more strenuous nature of the uphill modality. Compared with the first day, the exhaustive exercise time of the rats in each exercise group was drastically declined on day 7 (Figure 2B), accompanied by a significant decrease in body weight. Time to exhaustion was also lower in the U0 vs. D0 group, which is expected due to the more strenuous nature of uphill running.

### RBER Results in Morphological Alterations in Skeletal Muscle and Visceral Tissues

**FIGURE 3:**
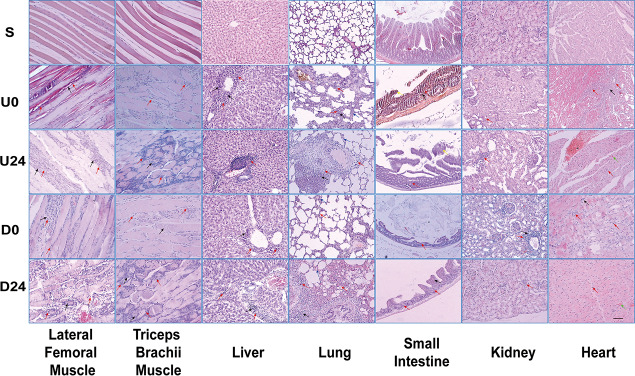
Light microscopy demonstrating morphologic changes in various tissues/organs after RBER. Arrows: Black = necrosis; Red = inflammatory infiltration; Green = microhemorrhage; Blue = alveolar septum thickening; Yellow = detached intestinal mucosa. S, sedentary; U0, uphill training, tissues collected immediately; U24, uphill, 24 h later; D0, downhill, immediately; D24, downhill, 24 h later. *n* ≥ 10. Scale bar = 100 μm.

We next investigated if and how RBER affects organ function by assessing for morphological changes in the collected tissues by H&E staining. Except for the sedentary group S in which the organs showed normal morphological appearance, the organs in all exhaustive exercise groups demonstrated varying degrees of morphological abnormality and infiltration of inflammatory cells such as neutrophils and macrophages (Figure 3 and Supplementary Figure S1). The morphological changes associated with each tissue type are elaborated below.

#### Skeletal Muscle

Compared with group S, skeletal muscle cells in each exercise group were larger, with unclear muscle fiber boundaries, pale cytoplasm, and uneven staining. Microhemorrhages are noted throughout the tissue from exhaustive exercise subjects. Compared with uphill running groups U0 and U24, rats in the downhill running groups D0 and D24 had more serious necrosis and the inflammatory infiltration of triceps brachii muscle while they had relatively less damage in the lateral femoral muscle.

#### Small Intestine

In U0 and D0 groups, the intestinal mucosa of rats appeared detached from the underlying tissue with shortened or lost villi, and the intestinal wall became notably thinner. In U24 and D24 groups, the intestinal wall became much thinner, and a large number of inflammatory cells gathered to form necrotic foci. The degree of lesion was higher in the downhill running groups than that in the uphill running groups.

#### Liver

Hepatic cell disorder, hepatic cord fibrosis, central vein congestion, inflammatory infiltration in the portal area, and focal necrosis were observed in all exercise groups. The degree of the hepatic lesions in the uphill running groups was higher than that in the downhill running groups.

#### Lung

Pulmonary alveolar swelling and rupture, thickening of the alveolar septum, blood vessel congestion, infiltration of inflammatory cells around blood vessels, and extensive necrosis of lung tissues were observed in all exercise groups. When compared with groups at the same observation time point, the degree of lung lesion in the downhill running group was higher than that in the uphill running group.

#### Kidney

Compared with group S, the kidney tissues in the downhill running groups were disorganized with signs of glomerular vacuolar degeneration. A large number of inflammatory cells were found infiltrating the perivascular areas and renal tubules. Occasional focal necrosis was also found. The above described abnormal renal morphology is especially prevalent in the downhill running groups, but not common in the uphill groups.

#### Heart

Compared with group S, inflammatory cell infiltration was observed in the hearts of all exercise groups. Focal necrosis, intravascular coagulation, and scattered interstitial hemorrhage were occasionally found. There was no significant difference in morphological changes between the two exercise modes.

### RBER Results in Blood and Immune Cell Changes Consistent With Systemic Inflammation

**FIGURE 4:**
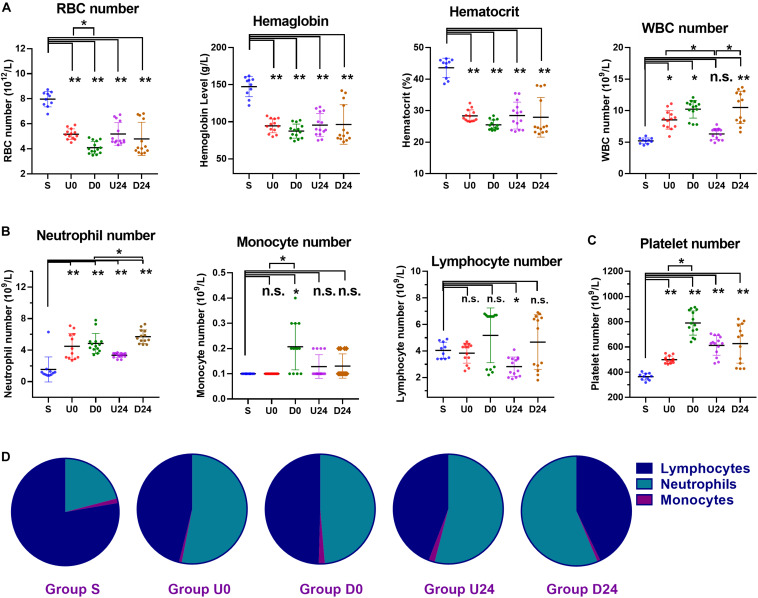
Changes in the numbers and percentages of blood cells in different groups. **(A)** Compared with sedentary group S, the hemoglobin level, hematocrit, and number of red blood cells (RBC) were all decreased in each exercise group, while white blood cells (WBCs) were increased. **(B)** The WBC differential demonstrated a significant increase in number of neutrophils in each exercise group. The number of monocytes was roughly unaffected, except in group D0 which demonstrated a significant increase. The number of lymphocytes was roughly unaffected, except in group U24 which demonstrated a significant decrease. **(C)** The platelet numbers were increased in all exercise groups. **(D)** The percentage of neutrophils was increased each exercise group (all *P* < 0.01). As a result, the percentage of lymphocytes was decreased in each exercise group (all *P* < 0.01). S, sedentary; U0, uphill training, samples collected immediately; U24, uphill, 24 h later; D0, downhill, immediately; D24, downhill, 24 h later. *P < 0.05; **P < 0.01, n.s., not significant. *n* ≥ 10.

To evaluate the cellular composition of blood during RBER, whole blood from the abdominal aorta of all groups underwent complete blood cell counting. We identified an overall decrease in number of erythrocytes, hemoglobin content, and the hematocrit of the rats in each exercise group compared with those in the S group (all *p* < 0.01). The number of erythrocytes in the D0 group was significantly lower than that in the U0 group (*p* < 0.05) (Figure 4A).

The white blood cell (WBC) group participates critically in immunological and inflammatory responses. Compared with group S, the WBC count in U0 (*p* < 0.05), D0 (*p* < 0.01), and D24 (*p* < 0.01) increased significantly. The WBC count in the U24 group was significantly lower than that of the U0 (*p* < 0.05) and D24 groups (*p* < 0.05). Specifically, neutrophils are a major leukocyte type that play a role as initial mediators of the inflammatory response. The number and percentage of neutrophils in the exercise groups were significantly higher than those in the S group (Figure 4B; all *p* < 0.01). Notably, this elevation persisted in both the U24 and D24 groups. Monocytes and lymphocytes are important immunoreactive regulators, with lymphocytes being the primary cells of the adaptive immune response. The number of monocytes in the D0 group was significantly higher than that in group S and U0. The percentage of monocytes in D0 group was significantly higher than that in groups D24 and U0 (both *p* < 0.05) (Figure 4D). The percentage of lymphocytes in each group was significantly lower than that in S group (*p* < 0.01) (Figure 4D).

The platelet is the regulator of blood coagulation. The platelet count in each exercise group was significantly higher than that in group S (*p* < 0.01), among which the platelet count in the D0 group was significantly higher than that of the U0 group (*p* < 0.05) (Figure 4C).

In summary, RBER in rats caused decreases in the number of erythrocytes, the content of hemoglobin, and the hematocrit, but increases in the number of total WBCs, the number and percentage of neutrophils, and the number of platelets. These alterations in the animals’ immune-related cells demonstrate an ongoing systemic inflammatory reaction that may underlie the multi-organ damages we observed.

### RBER Results in Elevations of Serum Enzymes and Activity

**FIGURE 5:**
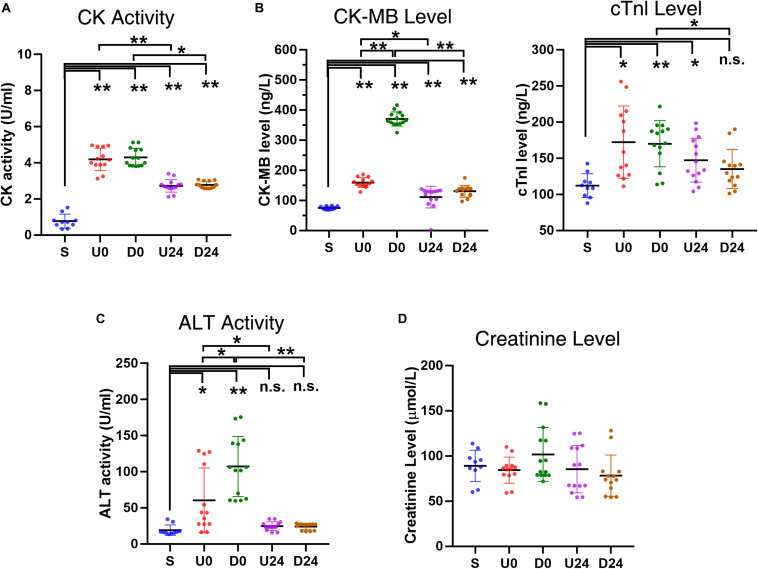
Changes in serum enzyme activities and levels after RBER. **(A)** Serum creatine kinase (CK) activity was greatly increased in each exercise group, reflecting skeletal muscle damage in the rats following exercise **(B)** Upregulation of specific myocardial marker CK-myocardial band (CK-MB) and cardiac troponin I (cTnI) in each exercise group, indicative of myocardial injury after exercise. The upregulation noted in U0 and D0 was largely recovered after 24 h of rest as shown in U24 and D24. **(C)** The serum alanine aminotransferase (ALT) activity was increased in U0 and D0, but not in U24 and D24, implying that damage of liver cells occurred acutely after exercise but was recovered after 24 h of rest. **(D)** The creatinine level was largely unaffected, indicating that the injury to kidney was minor in all exercise groups. S, sedentary; U0, uphill training, samples collected immediately; U24, uphill, 24 h later; D0, downhill, immediately; D24, downhill, 24 h later. *P < 0.05; **P < 0.01, n.s., not significant. *n* ≥ 10.

In addition to identifying morphologic alterations consistent with organ injury, damage caused by exhaustive exercise was also evaluated by changes in the serum levels and activities of specific enzymes, such as CK, ALT, CK-MB, and cTnI. CK is an enzyme found in the heart, brain, skeletal muscle, and other tissues with the small amount of CK normally in the blood being primarily derived from skeletal muscle. Increased amounts of CK are released into the blood when there is skeletal muscle and/or heart damage, and a drastic increase of CK usually indicates skeletal muscle damage. CK-MB and cTnI are more specific indicators of myocardial damage when their levels increase sharply. The serum CK activity in all exercise groups was 5-times higher than that in S group immediately and 24 h following exhaustive exercise (all *p* < 0.01, Figure 5A), consistent with skeletal muscle damage as described in previous studies. The CK activity declined significantly at 24 h after exercise in both the uphill and downhill groups (U0 vs. U24, *P* < 0.01; D0 vs. D24, *P* < 0.05). Similarly, the CK-MB activity in all exercise groups was significantly higher than that in group S (all *P* < 0.05, Figure 5B), and decreased significantly at 24 h after exercise (U0 vs. U24, *P* < 0.05; D0 vs. D24, *P* < 0.01). The serum cTnI levels groups followed a similar pattern with levels significantly higher than those in the S group (*P* < 0.05, *P* < 0.05, and *P* < 0.01 respectively), with its activity in both exercise modes being decreased at 24 h after exercise (Figure 5B). ALT is an enzyme found mostly in the cells of the liver, however, much smaller amounts of it are also found in the kidney, heart and muscles. Compared with group S, the activity of serum ALT increased significantly only immediately after exercise (U0 vs. S, *P* < 0.05; D0 vs. S, *P* < 0.01) (Figure 5C), and its activity in both exercise modes decreased significantly at 24 h after exercise (U0 vs. U24, *P* < 0.05; D0 vs. D24, *P* < 0.01). We also examined the serum levels of creatinine, an indicator of kidney function, and it does not seem to change in any of the groups (Figure 5D). The similar pattern of CK, CK-MB, cTnI and ALT indicates that RBER causes damage to organs such as skeletal muscles, heart and liver, but can be compensated for and alleviated by 24 h of rest.

### Serum Inflammatory Factors Are Upregulated by RBER

**FIGURE 6:**
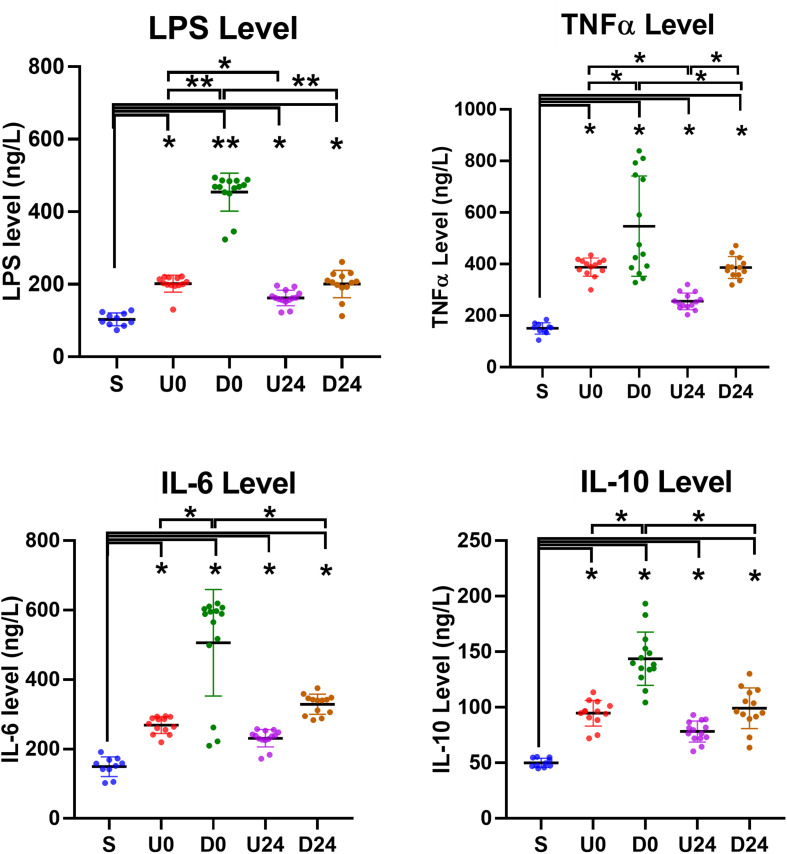
Serum changes in inflammation-related factors. The serum levels of lipopolysaccharide (LPS), tumor necrosis factor-alpha (TNF-α), interleukin (IL)-6 and IL-10 were all upregulated in each exercise group. They were relatively higher in downhill running groups than those in corresponding uphill groups. The degrees of increases were also significantly or insignificantly alleviated after 24 h of rest in U24 and D24 groups. S, sedentary; U0, uphill training, samples collected immediately; U24, uphill, 24 h later; D0, downhill, immediately; D24, downhill, 24 h later. *P < 0.05; **P < 0.01. *n* ≥ 10.

To further investigate the inflammatory changes caused by RBER at the molecular level, we examined the serum levels of LPS, TNFα, IL-6, and IL-10 in animals of all groups. The ELISA results showed that the serum concentrations of all four factors, LPS, TNFα, IL-6, and IL-10, were significantly higher in all exercise groups than those in group S (all *p* < 0.05, Figure 6), indicating that there were both elevated inflammatory and reactive anti-inflammatory responses in rats following exhaustive exercise. When the D24 group was compared with the D0 group, the concentrations of all four factors were significantly lower in D24 group, although still elevated. When the U24 group was compared with the U0 group, the concentrations of all four factors were also lower in U24 group, however, only the TNFα concentration was significantly lower by statistical analysis. Compared with the same observation points in different exercise modes, the serum level of LPS and TNFα were significantly higher in downhill groups than those in uphill groups (all *p* < 0.05). The IL-10 level in the D0 group was also significantly higher than that in the U0 group (*p* < 0.05). These data suggested that the inflammatory reactions were more severe in downhill running groups than in the corresponding uphill groups and, despite some signs of recovery, these inflammatory factors persist in an elevated state. These molecular findings may help us to understand the variation in morphologic, serum enzyme, and blood component alterations between the two modalities.

## Discussion

The pleiotropic effects of exercise on various organs systems of the body endorse it as a great tool to prolong life span and prevent and manage chronic disease. However, exhaustive exercise can induce stress beyond the body’s compensatory capacity leading to tissue damage and dysfunction in multiple organs and systems (Shephard, 2001; Hawley et al., 2014; Zierath and Wallberg-Henriksson, 2015). In this study, we have shown that, in addition to causing weight loss and decreased exercise capacity, repetitive exhaustive downhill and/or uphill running induces a sustained systemic inflammatory response, as demonstrated by extensive leukocytosis, elevated cytokines and inflammatory mediators, and infiltration of immune cells into various tissues including skeletal muscle, small intestine, liver, lung, kidney, and heart. The effect of exhaustive exercise on weight loss is consistent with previous reports, in humans, demonstrating weight loss within 3 days of training onset (Stawski et al., 2017). This exercise-induced systemic inflammation was further associated with serious morphological and/or functional alterations in skeletal muscle, small intestine, liver, and lung suggesting that exhaustive exercise may directly contribute to MOD.

Strenuous exercise leads to skeletal muscle damage via mechanical trauma as a result of the acute and repetitive mechanical traction placed directly on muscle fibers. Secondary inflammation of the muscle fibers and collagen, increased cell permeability, and microcirculatory impairment may further destroy muscle contraction components, leading to the release of muscle contents and necrosis (Malm, 2001). Consistent with these findings, we demonstrated that repeated uphill and downhill exhaustive exercises result in a loss of skeletal muscle fiber integrity, as demonstrated by the loss of clear boundaries and pale cytoplasmic staining, as well as infiltration of the skeletal muscle with inflammatory cells including neutrophils and macrophages and microhemorrhage. The morphological damage identified was further associated with elevated serum CK activity up to 5-fold in the U0/D0 groups and 3-fold in the U24/D24 groups as well as elevated levels of CK-MB. Both enzymes are released into the serum following muscle injury. It is well accepted that the mechanism of CK and CK-MB release is related to altered permeability of the myocyte membrane, possibly due to alterations in ion distribution or lack of ATP (Son et al., 2015). These mechanisms, including post-exercise inflammation, edema, and enzyme release are postulated to underlie the phenomenon of delayed-onset muscle soreness (DOMS), which describes the persistent pain and tenderness experienced by both elite and novice athletes several days following cessation of physical activity. The severity of DOMS can range from mild discomfort to severe, debilitating pain and is often worse with eccentric exercises, such as downhill running, in which there is lengthening of the muscle (Cheung et al., 2003). The higher occurrence of muscular damage in eccentric exercise is consistent with our findings of worsened muscular necrosis in the triceps brachii and significantly higher levels of CK-MB in the group exposed to downhill running.

Skeletal muscle damage is known to result in release of DAMPs, such as cell-free DNA into plasma, and can incite a systemic inflammatory response (Tug et al., 2015; Shockett et al., 2016). Long periods of intense exercise resulting in increased secretion of stress hormones, hyperthermia, oxidative stress, and massive release of various DAMPs is hypothesized to mobilize neutrophil (Shek and Shephard, 1998; Pedersen and Hoffman-Goetz, 2000) and platelet (Kestin et al., 1993; Couto et al., 2018) entry into the blood as part of exercise-induced inflammation. In this study we have shown that both uphill and downhill running result in leukocytosis, with the most significantly increased cell type being neutrophils. Platelet release following both types of exercise were also seen, with platelet count remaining elevated at 24 h following exercise cessation. This is consistent with previous work that has described activation of platelets following acute, intense exercise and may provide a mechanism underlying exercise-induced microcirculatory dysfunction (Heber and Volf, 2015). In addition to the cellular remodeling noted within the blood, we identified increased circulating levels of the pro-inflammatory factors LPS and TNF-alpha and the anti-inflammatory factors IL-6 and IL-10. Similar to the worsened damage of skeletal muscle in downhill running, the increase in inflammatory cells and factors above described were noted to be more dramatic in animals exposed to downhill running. This dose-response relationship between exercise intensity, muscle damage, and inflammatory markers supports the notion that skeletal muscle damage directly induces a systemic inflammatory response, likely through the persistent release of DAMPs. Interestingly, the neutrophilia induced by our model of exhaustive exercise also resulted in a relative decrease in the proportion of circulating lymphocytes. Lymphocytes, which include B-cells and T-cells, are components of the adaptive immune system and are critical in providing protection from both bacterial and viral pathogens. While the exhaustive exercise provoked an overall pro-inflammatory milieu, the dilution of lymphocytes might represent a mechanism underlying the stress-induced immunosuppression seen in endurance athletes during extreme regimens or competitions (Gunzer et al., 2012). The reduction in erythrocytes identified by this model of exhaustive exercise is consistent with previous reports of “sports anemia” in high performing athletes, although the mechanisms underlying it are not clear. The etiology of such sports anemia is likely multifactorial, with proposed causes including dilution of erythrocytes secondary to plasma volume expansion as a beneficial adaptation to exercise (Eichner, 1992), hemolysis due to mechanical destruction of erythrocytes in plantar vasculature (similar to marching hematuria) (Hallberg and Magnusson, 1984), and iron-deficiency attributable to increased iron demand, loss iron, or blockage of iron absorption by stress-induced hepcidin (Clenin et al., 2015). At physiologic levels, this anemia is compensated by stimulation of erythropoiesis and increased red blood cell mass, with resulting younger red blood cells having improved oxygen release and deformity that is beneficial for exercise (Mairbaurl, 2013), however, in the compromised individual or setting of exhaustive exercise, such anemia may result in decreased delivery of oxygen and subsequent hypoxia.

Systemic inflammation, such as that which we have described in response to muscle damage following exhaustive exercise, can result in significant damage to the distal tissues. The mechanisms by which systemic inflammation induces multi-organ injury and leads to MOD is perhaps most well understood in the setting of sepsis. In sepsis, invasion of pathogens provokes an immune response, and this immune response produces widespread alterations including multi-organ inflammatory cell infiltration, production of reactive oxygen species and subsequent apoptosis, and formation of microthrombi that disrupt microcirculation, ultimately leading to shock and death (Spapen et al., 2017). While rarely reported, MOD resulting from extreme physical exertion produces similar clinical manifestations as sepsis likely owing to related pathophysiologic processes (Zielinska-Borkowska et al., 2015). Our analysis of tissue from the heart, kidneys, lungs, liver, and small intestine reveals morphologic damage and dysfunction of these organs following exposure to repetitive exhaustive exercise. The observed changes in our model are not to the extent of those produced by such conditions as sepsis or trauma, where patients are often critically ill and require intensive cardiac, respiratory, and/or renal support. For example, the patient reported in above described case report had an altered level of consciousness, respiratory, and renal failure (Zielinska-Borkowska et al., 2015). However, the nature of the changes observed in animals, particularly the concordance of diffuse inflammatory and pathologic changes, is similar to that of the clinical syndrome of MOD suggesting that similar mechanisms underly these states. It would, therefore, also be interesting to determine if immunomodulatory therapy is able to attenuate the organ damage.

The cardiovascular response to exercise is well-described, with enhanced sympathetic activity resulting in increased heart rate (chronotropy) and enhanced contractility (inotropy) to provide increased cardiac output that matches the needs of the body (Laughlin, 1999). Further, in trained athletes, a chronic regimen of aerobic exercise induces physiologic cardiac remodeling, characterized by eccentric hypertrophy and improved left ventricular ejection fraction (Fernandes et al., 2015). However, acute exhaustive exercise has been associated with adverse cardiac responses including impaired cardiomyocyte Ca^2+^ handling and mitochondrial respiration, both of which are associated with dysfunction in systolic contraction and diastolic relaxation (Ljones et al., 2017), as well as lymphocytic infiltration and enhanced apoptotic signaling (Olah et al., 2015). In this study, we demonstrated only slight morphologic abnormalities of the heart, including focal areas of necrosis and interstitial hemorrhage, but substantial increases in the serum levels of CK-MB and cTnI, both of which are associated with cardiac muscle injury. These levels were significantly decreased at 24 h after exercise, indicative of a preserved compensatory ability of the heart. It is important to consider, though, that such a robust release of cardiac enzymes following 7 days of exercise may be indicative of a slowly progressive pathologic remodeling process that may manifest in individuals partaking in chronic exhaustive exercise regimens. Such pathologic remodeling has been reported in cases of chronic resistance exercise, in which persistent muscle damage may occur, and may be concerning for decline in cardiac function (Fernandes et al., 2015).

The heart and kidney play, arguably, the most important roles in multi-organ interaction, with their functional failure often marking the endpoint of MODS in the general population and as a severe sports complication. Acute kidney injury disrupts the body’s ability to filter and excrete substances from the blood, leading to accumulation of urea to toxic levels, metabolic imbalance of water and electrolytes, and disruption in normal acid-base status (Singbartl and Joannidis, 2015). All of these abnormalities result in systemic disturbances that induce and/or aggravate MOD (Lee et al., 2018). The occurrence of renal alterations in the exercise state, as evidenced by transient proteinuria, albuminuria, and hematuria in athletes, has been recognized for over 100 years (Collier, 1907). More recent work using renal biopsy and autopsy specimens has demonstrated that exhaustive exercise can lead to acute renal tubular obstruction and necrosis (Howenstine, 1960; Schrier et al., 1967). In this current study, while we identified conditions, such as exertional rhabdomyolysis and heat stress, which are known to affect renal function, we identified only slight morphological abnormality in the kidneys of each exercise group, and found no significant difference in the Cr level between the groups, suggesting that renal filtration was relatively normal across all exercise groups. This suggests that renal alterations are end-stage outcomes of exhaustive exercise that may manifest in more long-term or strenuous exercise. It is also important to consider that exhaustive exercise in populations with already reduced renal function may be at particular risk for accumulation of toxic metabolites.

Other tissues that demonstrated morphologic alterations in this study included the small intestine, which demonstrated shedding and necrosis of intestinal mucosa, and the liver, which had areas of focal necrosis and local inflammatory infiltrate in the parenchyma in addition to elevations in serum ALT. We hypothesize that such damage is secondary to ischemic and/or reperfusion injury that occurs when perfusion of the splanchnic bed is reduced in an effort to maintain blood flow to active skeletal muscle (Gleeson, 2000). The damage noted to the intestinal mucosa is particularly concerning as destruction of the intestinal mucosal barrier permits release of pathogen-associated molecular patterns (PAMPs) and DAMPs from the gut microbiota that may incite further inflammatory action (Lambert et al., 2002; Dokladny et al., 2006). In the respiratory tract, we have also described lung tissue changes including thickening of the alveolar septum, infiltration of inflammatory cells, and massive necrosis. We hypothesize that such damage is evidence of direct damage from the increased ventilation associated with exhaustive exercise as well as an example of the multi-organ injury resultant of sustained systemic inflammation. Similar to the intestine, exhaustive exercise may perturb the mucosal immunity of the respiratory tract and can contribute to subsequent infection, such as the upper respiratory tract infections known to afflict athletes (Gleeson, 2000). We do not have any evidence regarding the impact of exhaustive exercise on the microbiome itself, although previous studies have demonstrated a role for alterations in microbiome related to exercise-induced adaptations (Mach and Fuster-Botella, 2017). Within the lungs, changes in microbial diversity have been associated with chronic obstructive pulmonary disease exacerbations in association with changes in inflammatory factors (Wang et al., 2016). It is also known that pathologic changes in microbial diversity are associated with disruption of the gut-lumen barrier (Hayes et al., 2018). Therefore, it would be interesting to determine if microbiome changes underly the observed damage to the intestinal and respiratory mucosa. Areas of focal histological damage were scattered throughout the observed sections. However, this analysis was limited to similar regions within an organ and did not directly compare differential effects in different regions of the same organ. Future research employing exhaustive exercise models to study its effect on organ systems should consider regions-specific effects within an organ (e.g., left ventricle of heart vs. right).

We have here showed significant morphological, cellular, and biochemical changes following intense episodes of exhaustive exercise in rats. While many of the disturbances described as a result of RBER appear to be more or less transient, there is evidence of sustained inflammation at 24 h following the exhaustive exercise training. In comparing the results from animals sacrificed immediately versus 24 h post-exercise, there appears to be a sustained statistically significant increase in WBC count, neutrophils, LPS, and TNF-alpha consistent with a non-specific inflammatory response provoked by systemic insult, like exercise. This novel finding is in contrast to previous evidence that demonstrated that response of immune cells to acute exhaustive exercise is worsened by repetitive exercise and/or skeletal muscle injury, however, once the exercise regimen is completed, the change in leukocyte count quickly recovers and approaches pre-exercise levels within a period of several hours to one day (Weinstock et al., 1997). This highlights the notion that the pro-inflammatory changes associated with exercise are transient, while long-term exercise and conditioning are thought to be associated with an anti-inflammatory milieu (Ploeger et al., 2009; Sallam and Laher, 2016). It is possible that a longer duration of study in these animals would have resulted in transition to an anti-inflammatory response that is associated with muscle recovery and regeneration. However, the exhaustive nature of this training model, exceeding normal adaptive reserve, acts as a major insult to the system, and causes potentially irreparable damage. This may underlie the difference between the deleterious effects of exhaustive exercise and the beneficial effects of long-term exercise to which the animal is adapted. While some restoration of the pre-exercise milieu was noted, even after one day the inflammatory alterations remained. As such, the potential for systemic inflammation to persist and result in widespread damage, and MOD, may be a concern in scenarios involving exhaustive exercise or overtraining.

It is important to recognize the limitations of this study, particularly the limited time points of data collection. The available data only permit comparison between baseline (pre-exercise), immediately following exercise, and 24 h following exercise. As such, we do not have the ability to describe the timeframe regarding onset, development, and progression of inflammation and subsequent MOD. Previous work has demonstrated that a single bout of exhaustive exercise is capable of inducing release of cell free DNA, formation of reactive oxidative species, and acute phase inflammatory responses (Steinbacher and Eckl, 2015; Kaspar et al., 2016; Stawski et al., 2017). Therefore, it is reasonable to postulate that these may act as initiating events to trigger subsequent pathologic changes throughout the body. Unfortunately, we were unable to assay levels of cell free DNA or reactive oxidative species in this study to determine if our RBER model induces such changes. Future experiments focused on mechanistic underpinnings of the inflammatory response and organ changes observed here are certainly warranted. In particular, utilizing pharmacologic (i.e., corticosteroids, immunomodulators) or transgenic lines (i.e., deletion of specific cytokines) to dampen the inflammatory response would provide convincing evidence of the causal role of inflammation in organ pathology. Finally, while our interest was establishing evidence for sustained inflammation and pathologic changes using a novel model of exhaustive exercise, we did not determine long-term effects beyond 24 h. This would be an interesting undertaking that would permit an understanding of the timeframe of recovery, which is particularly clinically relevant, and the mechanisms mediating such recovery.

## Conclusion and Future Directions

Here we demonstrate that RBER results in serious injury to skeletal muscle that triggers a subsequent systemic inflammatory reaction that induces tissue necrosis and dysfunction. Furthermore, exercise-induced ischemia can cause intestinal barrier dysfunction that permits entry of DAMPs and PAMPs from the microbiota which can incite further immune response. Taken together, these multisystem alterations are consistent with features of such conditions as MOD. Moreover, as demonstrated by reduction in serum levels of tissue damage markers and repair of tissue, the organs demonstrate some compensatory capacities that help to protect the heart and kidney from severe destruction, which is often the end result of MOD and would be fatal. These results highlight the pathologic alterations of exhaustive exercise and provide insight into strategies that may prevent or treat sports-induced MOD, such as avoiding repetitive heavy-load exercises, improving exercise tolerance through progressive healthy training, and ensuring proper digestive health to maintain integrity of mucosal barriers and prevent microbiome dysbiosis.

## Data Availability Statement

The datasets generated for this study are available as Supplementary Material. Any further details are available on request to the corresponding author.

## Ethics Statement

The animal study was reviewed and approved by Committee for the Care and Use of Laboratory Animals at Tianjin University of Sport.

## Author Contributions

PL and HW conceived the study idea and designed the analysis. QH, XZ, HC, and QL conducted the experiments and analyzed the data. KM verified and optimized the analytical methods. JW helped to supervise the project. DQ contributed to the interpretation of the results. All authors discussed the results and contributed to the final version of the manuscript.

## Conflict of Interest

KM was employed by Jiangsu Biodep Biotechnology and Probiotics, Australia. JW was employed by Beijing Allwegene Health. DQ was employed by Beijing Tong Ren Tang Health-Pharmaceutical. The remaining authors declare that the research was conducted in the absence of any commercial or financial relationships that could be construed as a potential conflict of interest.
