# Supplementary material for: Repetitive Bouts of Exhaustive Exercise Induces a Systemic Inflammatory Response and Multi-Organ Damage in Rats
Source: Front Physiol. 2020 Jun 23;11:685. doi: 10.3389/fphys.2020.00685 (PMC7324715; doi:10.3389/fphys.2020.00685)
Supplement: Supplementary file 1 [file Data_Sheet_1.DOC]

# Figure 2

# A

## Body weight (g )

| S | Day 0 | 292 | 295 | 312 | 310 | 315 | 308 | 320 | 327 | 327 | 288 |  |  |  |  |
| --- | --- | --- | --- | --- | --- | --- | --- | --- | --- | --- | --- | --- | --- | --- | --- |
|  | Day 7 | 305 | 310 | 325 | 321 | 327 | 322 | 340 | 352 | 340 | 298 |  |  |  |  |
| U0 | Day 0 | 326 | 296 | 317 | 327 | 298 | 295 | 321 | 319 | 303 | 291 | 320 | 316 | 318 |  |
|  | Day 7 | 305 | 275 | 299 | 274 | 271 | 258 | 263 | 260 | 284 | 264 | 273 | 261 | 294 |  |
| D0 | Day 0 | 290 | 337 | 295 | 295 | 297 | 349 | 350 | 286 | 344 | 290 | 289 | 330 | 287 | 320 |
|  | Day 7 | 238 | 274 | 268 | 257 | 245 | 266 | 274 | 243 | 285 | 244 | 249 | 273 | 253 | 222 |
| U24 | Day 0 | 285 | 315 | 284 | 290 | 351 | 292 | 286 | 295 | 279 | 306 | 329 | 340 | 338 | 326 |
|  | Day 7 | 259 | 270 | 254 | 257 | 290 | 260 | 254 | 264 | 257 | 262 | 282 | 287 | 288 | 272 |
| D24 | Day 0 | 314 | 306 | 332 | 315 | 290 | 307 | 318 | 298 | 295 | 298 | 331 | 323 | 321 |  |
|  | Day 7 | 220 | 227 | 211 | 221 | 212 | 257 | 223 | 217 | 203 | 218 | 291 | 279 | 297 |  |

# B

## Exhaustive exercise time（min）

| U0 | Day 0 | 140 | 129 | 151 | 171 | 85 | 171 | 173 | 143 | 91 | 122 | 168 | 163 | 98 |  |
| --- | --- | --- | --- | --- | --- | --- | --- | --- | --- | --- | --- | --- | --- | --- | --- |
|  | Day 7 | 52 | 47 | 72 | 51 | 62 | 107 | 60 | 95 | 45 | 54 | 63 | 91 | 51 |  |
| D0 | Day 0 | 152 | 169 | 191 | 130 | 179 | 152 | 158 | 165 | 170 | 188 | 165 | 175 | 100 | 162 |
|  | Day 7 | 67 | 49 | 117 | 73 | 59 | 32 | 86 | 58 | 54 | 51 | 62 | 49 | 55 | 48 |
| U24 | Day 0 | 182 | 185 | 208 | 195 | 170 | 83 | 65 | 187 | 107 | 149 | 146 | 92 | 90 | 150 |
|  | Day 7 | 73 | 92 | 59 | 57 | 89 | 83 | 38 | 44 | 32 | 84 | 88 | 49 | 35 | 65 |
| D24 | Day 0 | 138 | 201 | 207 | 171 | 158 | 161 | 139 | 199 | 146 | 116 | 120 | 127 | 140 | 138 |
|  | Day 7 | 101 | 55 | 35 | 60 | 50 | 91 | 53 | 58 | 56 | 54 | 43 | 65 | 50 | 101 |

# Figure 4

# A

## RBC number（10^12^/L）

| S | 8.23 | 8.74 | 6.78 | 7.55 | 8.39 | 7.38 | 8.54 | 7.68 | 7.95 | 8.45 |  |  |  |  |
| --- | --- | --- | --- | --- | --- | --- | --- | --- | --- | --- | --- | --- | --- | --- |
| U0 | 5.14 | 5.03 | 4.87 | 5.01 | 4.5 | 5.31 | 5.21 | 4.76 | 5.61 | 5.79 | 4.75 | 5.91 | 5.36 |  |
| D0 | 3.53 | 3.81 | 3.8 | 3.49 | 3.69 | 3.63 | 4.3 | 4 | 3.95 | 4.9 | 4.79 | 4.75 | 4.09 | 4.64 |
| U24 | 4.53 | 4.64 | 4.58 | 6.59 | 5.44 | 6.73 | 4.83 | 4.64 | 4.54 | 4.45 | 4.31 | 6.24 | 4.69 | 6.46 |
| D24 | 4.09 | 4.03 | 3.87 | 4.61 | 3.71 | 3.63 | 4.19 | 6.41 | 6.75 | 3.86 | 6.68 | 3.68 | 6.82 |  |

## Hemoglobin（g/L）

| S | 144 | 155 | 122 | 129 | 139 | 160 | 157 | 156 | 150 | 162 |  |  |  |  |
| --- | --- | --- | --- | --- | --- | --- | --- | --- | --- | --- | --- | --- | --- | --- |
| U0 | 83 | 94 | 105 | 82 | 90 | 80 | 108 | 98 | 104 | 97 | 94 | 89 | 105 |  |
| D0 | 94 | 89 | 100 | 76 | 102 | 74 | 86 | 101 | 82 | 90 | 91 | 78 | 83 | 78 |
| U24 | 89 | 76 | 87 | 114 | 100 | 120 | 99 | 83 | 90 | 115 | 75 | 89 | 118 | 83 |
| D24 | 81 | 85 | 73 | 78 | 75 | 77 | 139 | 78 | 132 | 89 | 142 | 122 | 82 |  |

## Hematocrit（%）

| S | 43.8 | 45.4 | 46 | 45 | 46.1 | 38.5 | 40.2 | 45.2 | 46.5 | 39.3 |  |  |  |  |
| --- | --- | --- | --- | --- | --- | --- | --- | --- | --- | --- | --- | --- | --- | --- |
| U0 | 29.4 | 32.4 | 26.5 | 27.6 | 26.7 | 27.1 | 28.5 | 31.1 | 28.3 | 30.2 | 26.7 | 26.8 | 27.2 |  |
| D0 | 25.3 | 27.6 | 24.3 | 23.8 | 25.4 | 23.7 | 27.1 | 26 | 27.3 | 25.4 | 28.4 | 24.3 | 24.6 | 24 |
| U24 | 23.8 | 29.6 | 23.8 | 23.4 | 35.4 | 29.3 | 33.4 | 26.6 | 35.5 | 26 | 31.1 | 30 | 26.8 | 23.8 |
| D24 | 24.8 | 24.6 | 24.2 | 22.6 | 24.9 | 23.2 | 37.8 | 33.2 | 38.1 | 23.1 | 38 | 25.1 | 23.4 |  |

## WBC number（10^9^/L）

| S | 5.3 | 5.6 | 4.8 | 5.6 | 5.1 | 5.3 | 4.7 | 4.5 | 5.2 | 6 |  |  |  |  |
| --- | --- | --- | --- | --- | --- | --- | --- | --- | --- | --- | --- | --- | --- | --- |
| U0 | 10.7 | 7.1 | 11.1 | 7.4 | 9.7 | 7.5 | 9.4 | 8.4 | 8.9 | 7.6 | 8.7 | 5.9 | 8.7 |  |
| D0 | 11 | 11.4 | 11.5 | 9.9 | 10.6 | 12.1 | 10.7 | 10.1 | 10.2 | 11 | 7.8 | 7.8 | 11.2 | 8 |
| U24 | 6.9 | 4.9 | 7 | 6.2 | 6.9 | 6 | 6.9 | 5.7 | 5.3 | 5.3 | 7.1 | 7.8 | 5.3 | 6.9 |
| D24 | 12.6 | 13.7 | 11.5 | 8.9 | 12.8 | 8.4 | 7.3 | 13.1 | 13.4 | 6.6 | 11.7 | 8.2 | 8.5 |  |

# B

## Neutrophil number（10^9^/L）

| S | 0.9 | 0.8 | 1.2 | 0.8 | 1.6 | 1 | 1.2 | 0.9 | 1.3 | 1.1 | 6.3 |  |  |  |
| --- | --- | --- | --- | --- | --- | --- | --- | --- | --- | --- | --- | --- | --- | --- |
| U0 | 3.1 | 6.8 | 2.7 | 7.1 | 3 | 5.9 | 3.6 | 5.4 | 3.1 | 4.2 | 2.9 | 6 |  |  |
| D0 | 4.1 | 4.5 | 4.7 | 7.3 | 3.6 | 5.1 | 3.9 | 7.8 | 3.4 | 4.2 | 4.8 | 5.1 | 4.3 | 5.1 |
| U24 | 3.3 | 2.8 | 3.3 | 3.6 | 3.5 | 3.6 | 3.7 | 3.6 | 2.8 | 3.1 | 3.6 | 3.5 | 3.2 | 3.3 |
| D24 | 5.5 | 7 | 4.8 | 5.9 | 6.4 | 5.3 | 4.7 | 7.3 | 6.5 | 4.7 | 5.2 | 5.8 | 5.2 |  |

## Monocyte number（10^9^/L）

| S | 0.1 | 0.1 | 0.1 | 0.1 | 0.1 | 0.1 | 0.1 | 0.1 | 0.1 | 0.1 |  |  |  |  |
| --- | --- | --- | --- | --- | --- | --- | --- | --- | --- | --- | --- | --- | --- | --- |
| U0 | 0.1 | 0.1 | 0.1 | 0.1 | 0.1 | 0.1 | 0.1 | 0.1 | 0.1 | 0.1 | 0.1 | 0.1 | 0.1 |  |
| D0 | 0.3 | 0.3 | 0.2 | 0.2 | 0.2 | 0.4 | 0.2 | 0.1 | 0.1 | 0.1 | 0.2 | 0.1 | 0.2 | 0.3 |
| U24 | 0.1 | 0.2 | 0.2 | 0.1 | 0.1 | 0.1 | 0.1 | 0.1 | 0.1 | 0.1 | 0.1 | 0.2 | 0.1 | 0.2 |
| D24 | 0.2 | 0.1 | 0.2 | 0.1 | 0.1 | 0.1 | 0.1 | 0.1 | 0.1 | 0.1 | 0.1 | 0.2 | 0.2 |  |

## Lymphocyte number （10^9^/L）

| S | 4.3 | 4.9 | 3.5 | 4.7 | 3.4 | 4.2 | 3.4 | 3.4 | 3.8 | 4.8 |  |  |  |  |
| --- | --- | --- | --- | --- | --- | --- | --- | --- | --- | --- | --- | --- | --- | --- |
| U0 | 4.3 | 3.8 | 4.3 | 4.5 | 2.5 | 4.4 | 3.5 | 4.7 | 3.5 | 4.3 | 4.5 | 2.9 | 2.7 |  |
| D0 | 6.6 | 6.6 | 6.6 | 2.4 | 6.8 | 6.6 | 6.6 | 2.2 | 6.7 | 6.7 | 2.8 | 2.6 | 6.7 | 2.6 |
| U24 | 3.5 | 1.9 | 3.5 | 2.5 | 3.3 | 2.3 | 3.1 | 2 | 2.4 | 2.1 | 3.4 | 4.1 | 2 | 3.4 |
| D24 | 6.9 | 6.6 | 6.5 | 2.9 | 6.3 | 3 | 2.5 | 5.7 | 6.8 | 1.8 | 6.4 | 2.2 | 3.1 |  |

# C

## Platelet number （10^9^/L）

| S | 378 | 369 | 344 | 406 | 336 | 352 | 319 | 392 | 366 | 387 |  |  |  |  |
| --- | --- | --- | --- | --- | --- | --- | --- | --- | --- | --- | --- | --- | --- | --- |
| U0 | 512 | 495 | 470 | 504 | 470 | 546 | 502 | 489 | 532 | 463 | 550 | 486 | 477 |  |
| D0 | 730 | 663 | 898 | 906 | 789 | 755 | 671 | 774 | 893 | 872 | 739 | 915 | 640 | 820 |
| U24 | 604 | 470 | 629 | 705 | 638 | 629 | 562 | 514 | 480 | 679 | 649 | 629 | 696 | 697 |
| D24 | 806 | 680 | 772 | 470 | 604 | 429 | 508 | 785 | 822 | 692 | 428 | 431 | 727 |  |

# D

## Neutrophil（%）

| S | 16.98 | 14.29 | 25 | 14.29 | 31.37 | 18.87 | 25.53 | 20 | 25 | 18.33 | 16.98 | 14.29 | 25 | 14.29 |
| --- | --- | --- | --- | --- | --- | --- | --- | --- | --- | --- | --- | --- | --- | --- |
| U0 | 58.88 | 43.66 | 61.26 | 36.49 | 73.2 | 40 | 62.77 | 42.86 | 60.67 | 40.79 | 48.28 | 49.15 | 68.97 | 58.88 |
| D0 | 37.27 | 39.47 | 40.87 | 73.74 | 33.96 | 42.15 | 36.45 | 77.23 | 33.33 | 38.18 | 61.54 | 65.38 | 38.39 | 63.75 |
| U24 | 47.83 | 57.14 | 47.14 | 58.06 | 50.72 | 60 | 53.62 | 63.16 | 52.83 | 58.49 | 50.7 | 44.87 | 60.38 | 47.83 |
| D24 | 43.65 | 51.09 | 41.74 | 66.29 | 50 | 63.1 | 64.38 | 55.73 | 48.51 | 71.21 | 44.44 | 70.73 | 61.18 | 43.65 |

## Monocyte（%）

| S | 1.89 | 1.79 | 2.08 | 1.79 | 1.96 | 1.89 | 2.13 | 2.22 | 1.92 | 1.67 |  |  |  |  |
| --- | --- | --- | --- | --- | --- | --- | --- | --- | --- | --- | --- | --- | --- | --- |
| U0 | 0.93 | 1.41 | 0.9 | 1.35 | 1.03 | 1.33 | 1.06 | 1.19 | 1.12 | 1.32 | 1.15 | 1.69 | 1.15 |  |
| D0 | 2.73 | 2.63 | 1.74 | 2.02 | 1.89 | 3.31 | 1.87 | 0.99 | 0.98 | 0.91 | 2.56 | 1.28 | 1.79 | 3.75 |
| U24 | 1.45 | 4.08 | 2.86 | 1.61 | 1.45 | 1.67 | 1.45 | 1.75 | 1.89 | 1.89 | 1.41 | 2.56 | 1.89 | 2.9 |
| D24 | 1.59 | 0.73 | 1.74 | 1.12 | 0.78 | 1.19 | 1.37 | 0.76 | 0.75 | 1.52 | 0.85 | 2.44 | 2.35 |  |

## Lymphocyte（%）

| S | 81.13 | 87.5 | 72.92 | 83.93 | 66.67 | 79.25 | 72.34 | 75.56 | 73.08 | 80 |  |  |  |  |
| --- | --- | --- | --- | --- | --- | --- | --- | --- | --- | --- | --- | --- | --- | --- |
| U0 | 40.19 | 53.52 | 38.74 | 60.81 | 25.77 | 58.67 | 37.23 | 55.95 | 39.33 | 56.58 | 51.72 | 49.15 | 31.03 |  |
| D0 | 60 | 57.89 | 57.39 | 24.24 | 64.15 | 54.55 | 61.68 | 21.78 | 65.69 | 60.91 | 35.9 | 33.33 | 59.82 | 32.5 |
| U24 | 50.72 | 38.78 | 50 | 40.32 | 47.83 | 38.33 | 44.93 | 35.09 | 45.28 | 39.62 | 47.89 | 52.56 | 37.74 | 49.28 |
| D24 | 54.76 | 48.18 | 56.52 | 32.58 | 49.22 | 35.71 | 34.25 | 43.51 | 50.75 | 27.27 | 54.7 | 26.83 | 36.47 |  |

# Figure 5

# A

## CK（U/ml）

| S | 0.57 | 0.58 | 0.72 | 0.72 | 0.34 | 0.35 | 0.81 | 0.81 | 1.53 | 1.32 |  |  |  |  |
| --- | --- | --- | --- | --- | --- | --- | --- | --- | --- | --- | --- | --- | --- | --- |
| U0 | 4 | 4.37 | 4.9 | 4.9 | 3.91 | 3.89 | 4.95 | 4.97 | 4.17 | 4.22 | 3.25 | 3.13 | 3.89 |  |
| D0 | 4.73 | 4.71 | 5.13 | 5.13 | 3.84 | 3.83 | 3.85 | 3.85 | 4 | 4 | 4.27 | 4.27 | 4.83 | 3.81 |
| U24 | 2.79 | 2.81 | 2.17 | 2.13 | 2.62 | 2.61 | 3.31 | 3.4 | 2.65 | 2.65 | 2.8 | 2.59 | 2.7 | 2.91 |
| D24 | 3.01 | 3.02 | 2.65 | 2.65 | 2.59 | 2.6 | 2.8 | 2.79 | 3.05 | 3.08 | 2.58 | 2.58 | 2.76 |  |

# B

## CKMB（ng/L）

| S | 82.7 | 69.5 | 72.9 | 81.8 | 79.8 | 71.3 | 68 | 69.7 | 75.2 | 81.9 |  |  |  |  |
| --- | --- | --- | --- | --- | --- | --- | --- | --- | --- | --- | --- | --- | --- | --- |
| U0 | 144.5 | 153.1 | 149 | 128 | 156.3 | 152.4 | 163.4 | 150.9 | 179.1 | 180.3 | 161.5 | 167.5 | 186.7 |  |
| D0 | 416.7 | 355.2 | 359.8 | 353.2 | 385.4 | 382 | 359.2 | 404.1 | 364.4 | 392.1 | 325.4 | 353.6 | 366.3 | 373.4 |
| U24 | 120.2 | 103.6 | 129 | 138.4 | 82.9 | 88.5 | 132.4 | 2 | 133.1 | 131.5 | 128.6 | 130.2 | 130.3 | 104 |
| D24 | 141.3 | 135.9 | 138 | 139.1 | 141.6 | 138.1 | 96.7 | 175.3 | 119.4 | 103.8 | 119.9 | 131.2 | 117.3 |  |

## cTnI（ng/L）

| S | 95.83 | 118.06 | 112.5 | 98.61 | 116.58 | 142.89 | 131.94 | 108.68 | 108.61 | 87.94 |  |  |  |  |
| --- | --- | --- | --- | --- | --- | --- | --- | --- | --- | --- | --- | --- | --- | --- |
| U0 | 209.72 | 256.05 | 140.28 | 215.28 | 111.32 | 248.61 | 126.39 | 158.68 | 187.63 | 200.79 | 121.84 | 137.63 | 127.11 |  |
| D0 | 184.72 | 113.95 | 170.83 | 221.84 | 115.28 | 195.83 | 187.46 | 165.28 | 168.06 | 156.94 | 187.5 | 129.21 | 190.28 | 195.53 |
| U24 | 133.95 | 104.17 | 174.47 | 179.74 | 129.17 | 190.26 | 126.39 | 166.58 | 117.63 | 198.61 | 145.53 | 109.72 | 156.05 | 132.37 |
| D24 | 112.52 | 124.47 | 145.83 | 119.21 | 101.39 | 139.72 | 129.74 | 140.26 | 184.72 | 141.39 | 123.61 | 104.17 | 190.28 |  |

# C

## ALT（U/ml）

| S | 14.6 | 34.5 | 17 | 15.6 | 18 | 16.4 | 13.7 | 31 | 16.9 | 15.6 |  |  |  |  |
| --- | --- | --- | --- | --- | --- | --- | --- | --- | --- | --- | --- | --- | --- | --- |
| U0 | 27.8 | 43.5 | 27.6 | 16.6 | 129 | 124.8 | 27.9 | 43.8 | 35.6 | 16.7 | 127.4 | 110.7 | 54.5 |  |
| D0 | 60.7 | 62.7 | 173.6 | 101.9 | 139.4 | 106.6 | 59.9 | 60.4 | 175.8 | 102 | 138.3 | 106.5 | 143.9 | 70.4 |
| U24 | 23.6 | 23.6 | 28 | 34.9 | 15.9 | 23.8 | 23.7 | 23.6 | 28 | 34.9 | 16 | 23.8 | 30.6 | 19.5 |
| D24 | 17.9 | 27.3 | 27.3 | 26.7 | 27.3 | 17.5 | 18.3 | 27.4 | 27.2 | 26.8 | 28.9 | 18.5 | 25.2 |  |

# D

## Cr（μmol/L）

| S | 86.4 | 93.6 | 97.7 | 108.7 | 60.1 | 62.4 | 84.4 | 95.5 | 113.8 | 87.9 |  |  |  |  |
| --- | --- | --- | --- | --- | --- | --- | --- | --- | --- | --- | --- | --- | --- | --- |
| U0 | 59.2 | 105.5 | 85.2 | 85.6 | 87.5 | 89.2 | 60.1 | 110.1 | 80.1 | 79.4 | 86.6 | 89.4 | 78.6 |  |
| D0 | 157.8 | 82.4 | 94.5 | 78.8 | 117 | 78.8 | 158.6 | 82.4 | 95.3 | 79 | 117.4 | 78.8 | 131.7 | 71.9 |
| U24 | 66.9 | 67.3 | 124.7 | 54.2 | 108.5 | 90 | 67.1 | 67.5 | 125.3 | 54.4 | 108.9 | 90.5 | 59.3 | 111.6 |
| D24 | 82.8 | 73.5 | 64.4 | 54.4 | 54.7 | 77.7 | 83 | 73.9 | 55.3 | 128 | 70.5 | 77.9 | 120.6 |  |

# Figure 6

## LPS（ng/L）

| S | 97 | 95.9 | 118.8 | 128.3 | 73.9 | 85.7 | 123.8 | 112.2 | 89.7 | 107.3 |  |  |  |  |
| --- | --- | --- | --- | --- | --- | --- | --- | --- | --- | --- | --- | --- | --- | --- |
| U0 | 197.7 | 197.7 | 199.1 | 218.7 | 202.9 | 130.4 | 221.9 | 217.3 | 220 | 200.6 | 206.2 | 213.5 | 195.5 |  |
| D0 | 465 | 485.4 | 453.4 | 473.8 | 469 | 488.6 | 486 | 345.6 | 468.5 | 484 | 323.6 | 469.2 | 450.1 | 494.4 |
| U24 | 184.5 | 166.9 | 156.2 | 193.4 | 174 | 161.9 | 196.4 | 162.3 | 125 | 122.6 | 162.8 | 159.2 | 152.3 | 156.9 |
| D24 | 198.1 | 204.4 | 191.2 | 221.9 | 205.5 | 230.8 | 145.5 | 112.2 | 228.3 | 261.5 | 193.3 | 210.1 | 207.4 |  |

## TNFα（ng/L）

| S | 141.8 | 154.8 | 134.2 | 185.2 | 166.6 | 154 | 105.5 | 171 | 140 | 156.3 |  |  |  |  |
| --- | --- | --- | --- | --- | --- | --- | --- | --- | --- | --- | --- | --- | --- | --- |
| U0 | 407.4 | 418.4 | 350.9 | 365 | 300.1 | 401.4 | 389.5 | 435.6 | 397.4 | 415.5 | 411.7 | 375.3 | 378.4 |  |
| D0 | 839.9 | 590.9 | 746.3 | 392.8 | 363.5 | 386.6 | 344.6 | 425.7 | 328.5 | 475.2 | 438.9 | 728.5 | 810.1 | 793.1 |
| U24 | 262.9 | 241.5 | 257.4 | 294.8 | 235.3 | 321.1 | 295.9 | 239.3 | 220.4 | 230.9 | 203.8 | 276 | 249.1 | 253.5 |
| D24 | 372.3 | 388.6 | 358.9 | 444.2 | 472.2 | 319.2 | 358.9 | 375.2 | 403.2 | 335.9 | 390.2 | 387 | 425 |  |

## IL-6（ng/L）

| S | 142.6 | 159.1 | 141.6 | 169.1 | 158 | 102.2 | 105.7 | 173.1 | 151.9 | 191.5 |  |  |  |  |
| --- | --- | --- | --- | --- | --- | --- | --- | --- | --- | --- | --- | --- | --- | --- |
| U0 | 255.7 | 260.2 | 289.2 | 241.7 | 295.3 | 292.9 | 219.9 | 286 | 240.8 | 293.5 | 273 | 285.9 | 264.3 |  |
| D0 | 262.4 | 619.9 | 499.2 | 222.6 | 588.8 | 209.6 | 611.1 | 603.9 | 607.7 | 589.6 | 595.8 | 597.6 | 565.9 | 517.5 |
| U24 | 230.2 | 225.9 | 226.7 | 238.7 | 183.5 | 255.8 | 248.4 | 172.2 | 257.9 | 253.1 | 242.3 | 228.7 | 236.7 | 238 |
| D24 | 306 | 340 | 342.2 | 346.2 | 359.1 | 375.7 | 340.3 | 311.7 | 342.3 | 348.1 | 295.2 | 283.7 | 288.1 |  |

## IL-10（ng/L）

| S | 49.7 | 45.9 | 45.1 | 52.9 | 55.1 | 54.9 | 55.6 | 47.5 | 47.9 | 47.2 |  |  |  |  |
| --- | --- | --- | --- | --- | --- | --- | --- | --- | --- | --- | --- | --- | --- | --- |
| U0 | 91 | 94.3 | 96.5 | 105.3 | 113.6 | 106.6 | 72.1 | 96.6 | 75.1 | 94.8 | 96.8 | 88.6 | 101.4 |  |
| D0 | 148.9 | 161.3 | 153.1 | 114.8 | 135.3 | 104.4 | 127 | 138.6 | 193.5 | 135.2 | 134.1 | 144.7 | 139.9 | 183.2 |
| U24 | 79.6 | 76.6 | 73.2 | 64.6 | 81.9 | 88.9 | 60.5 | 72.2 | 93.2 | 89.2 | 76.5 | 72.1 | 81.6 | 86.7 |
| D24 | 103.8 | 115.8 | 89.7 | 95.1 | 130.3 | 105.5 | 96.3 | 73 | 119.3 | 63.8 | 91.7 | 113.3 | 93.9 |  |
