# Supplementary figures and images for: Repetitive Bouts of Exhaustive Exercise Induces a Systemic Inflammatory Response and Multi-Organ Damage in Rats
Source: Front Physiol. 2020 Jun 23;11:685. doi: 10.3389/fphys.2020.00685 (PMC7324715; doi:10.3389/fphys.2020.00685)

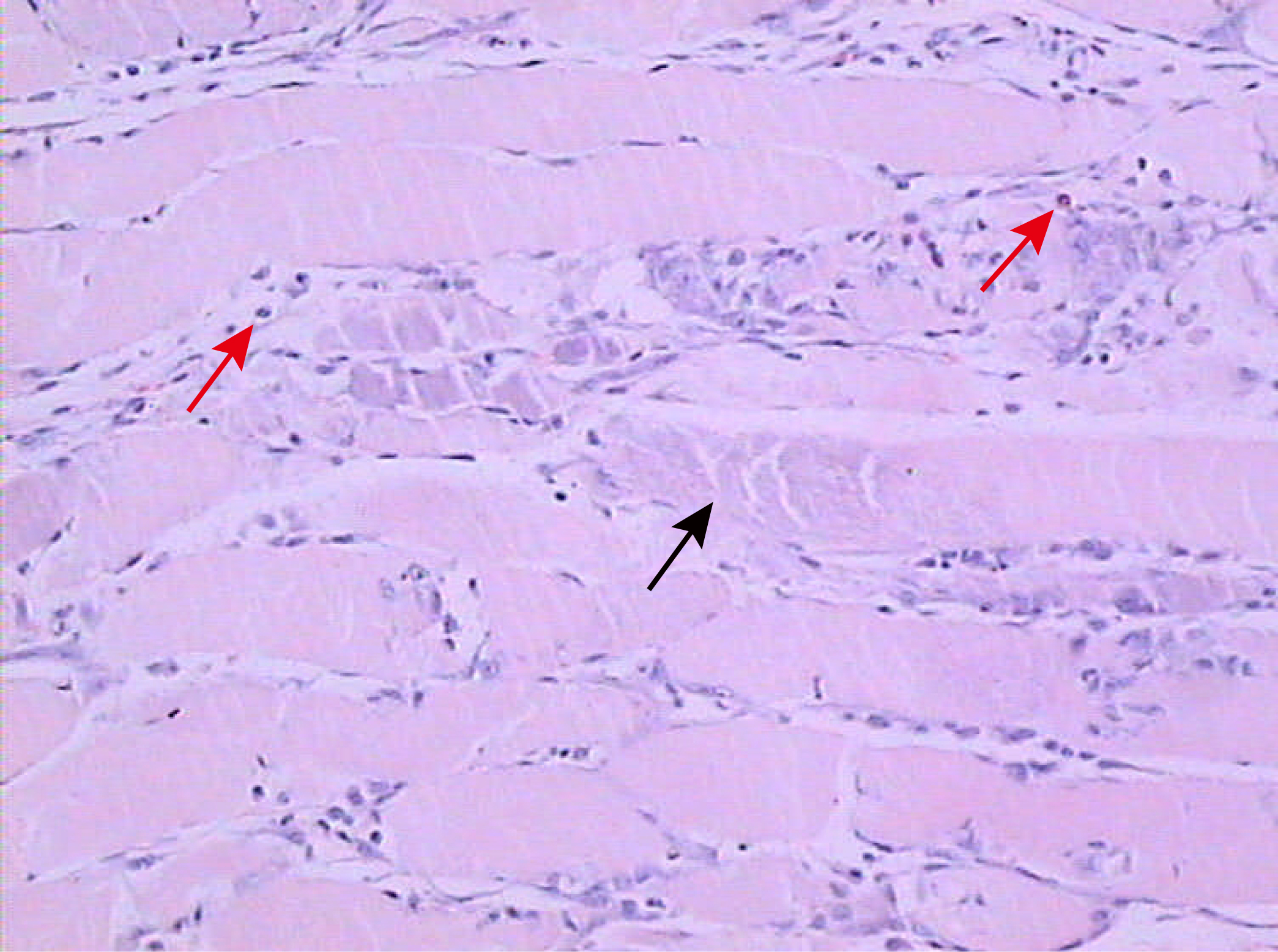

Supplement: Supplementary file 2 [file Data_Sheet_2.ZIP › Triceps Brachii Muscle/D0.jpg]

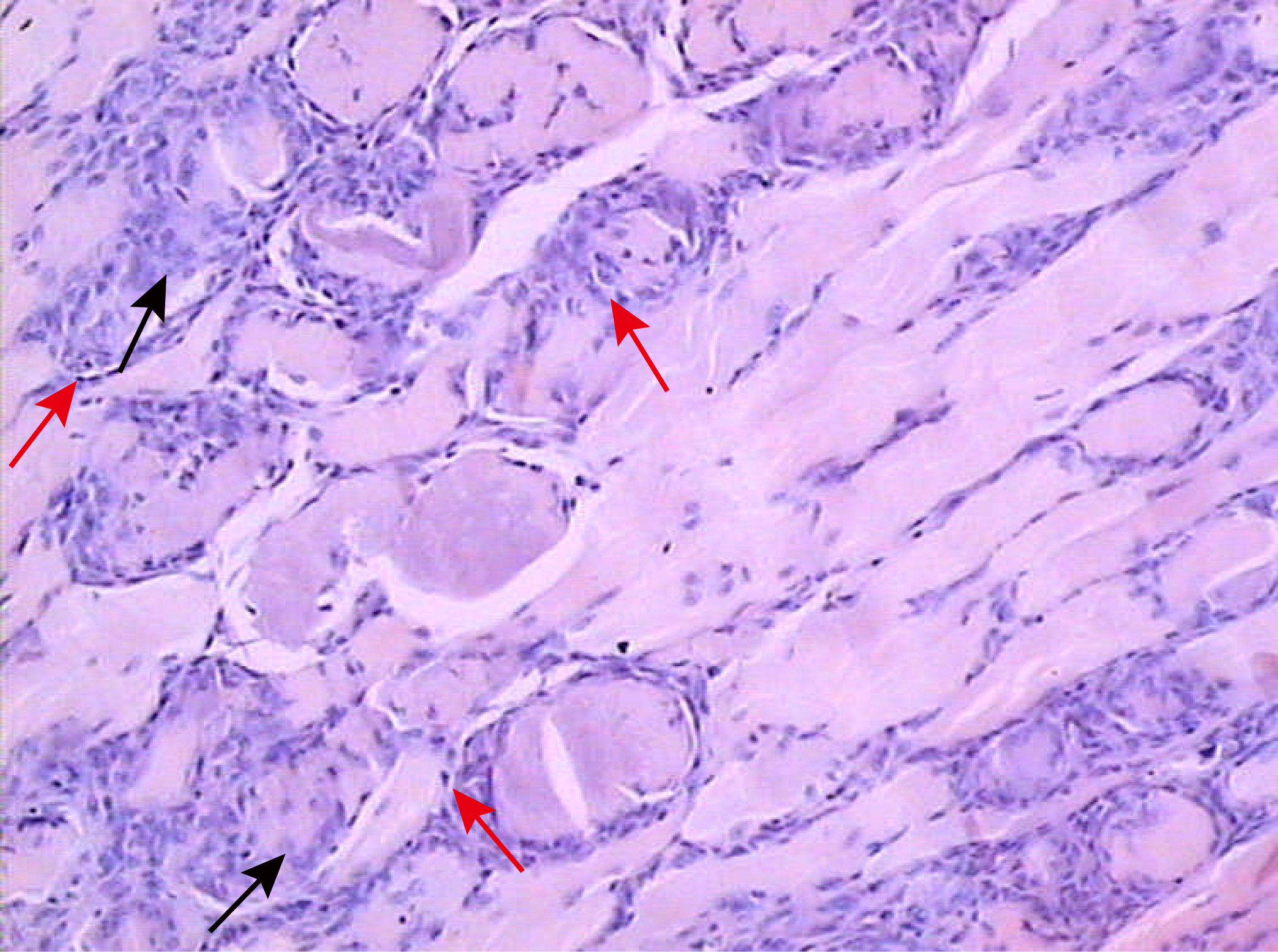

Supplement: Supplementary file 2 [file Data_Sheet_2.ZIP › Triceps Brachii Muscle/D24.jpg]

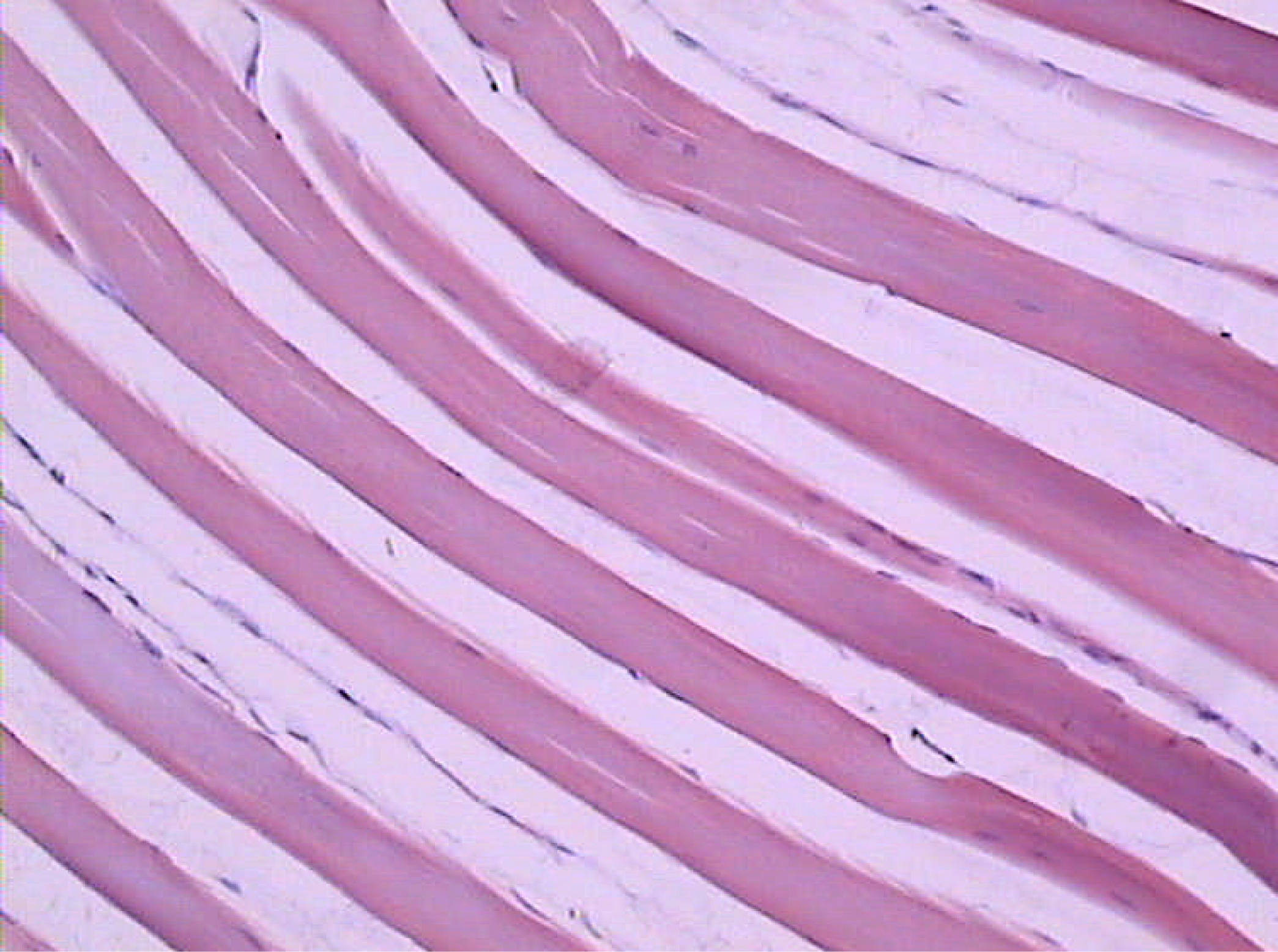

Supplement: Supplementary file 2 [file Data_Sheet_2.ZIP › Triceps Brachii Muscle/S.jpg]

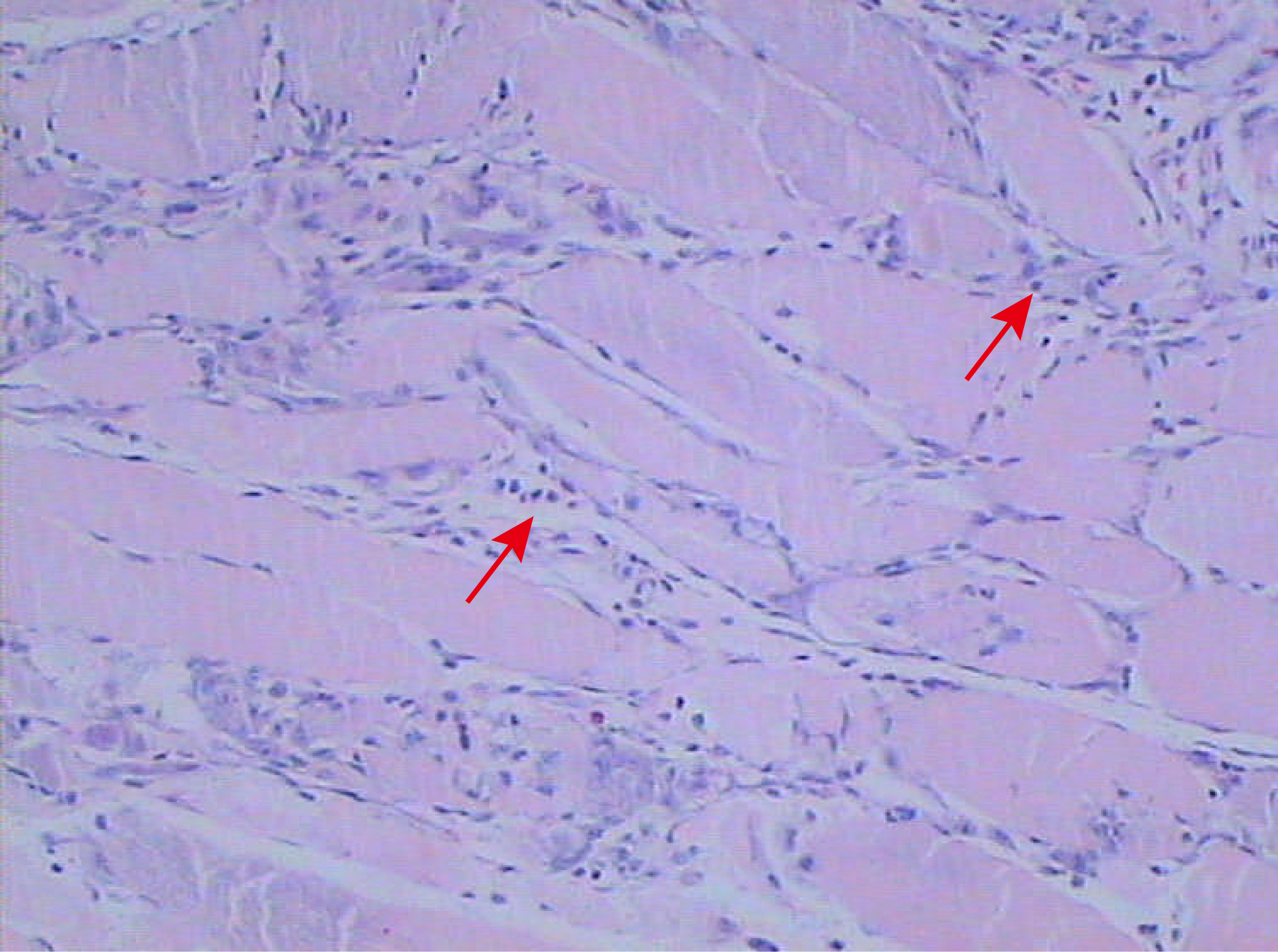

Supplement: Supplementary file 2 [file Data_Sheet_2.ZIP › Triceps Brachii Muscle/U0.jpg]

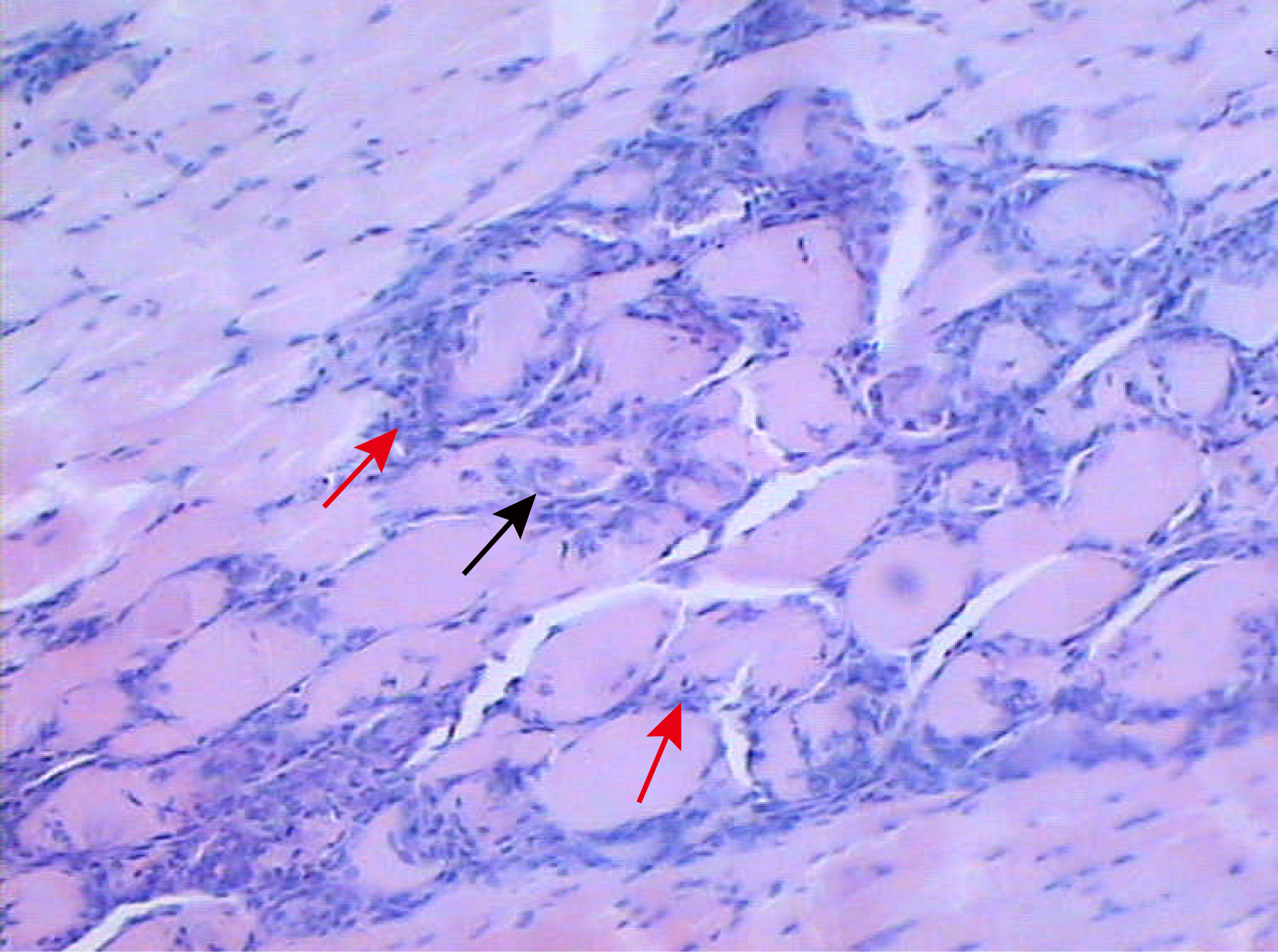

Supplement: Supplementary file 2 [file Data_Sheet_2.ZIP › Triceps Brachii Muscle/U24.jpg]

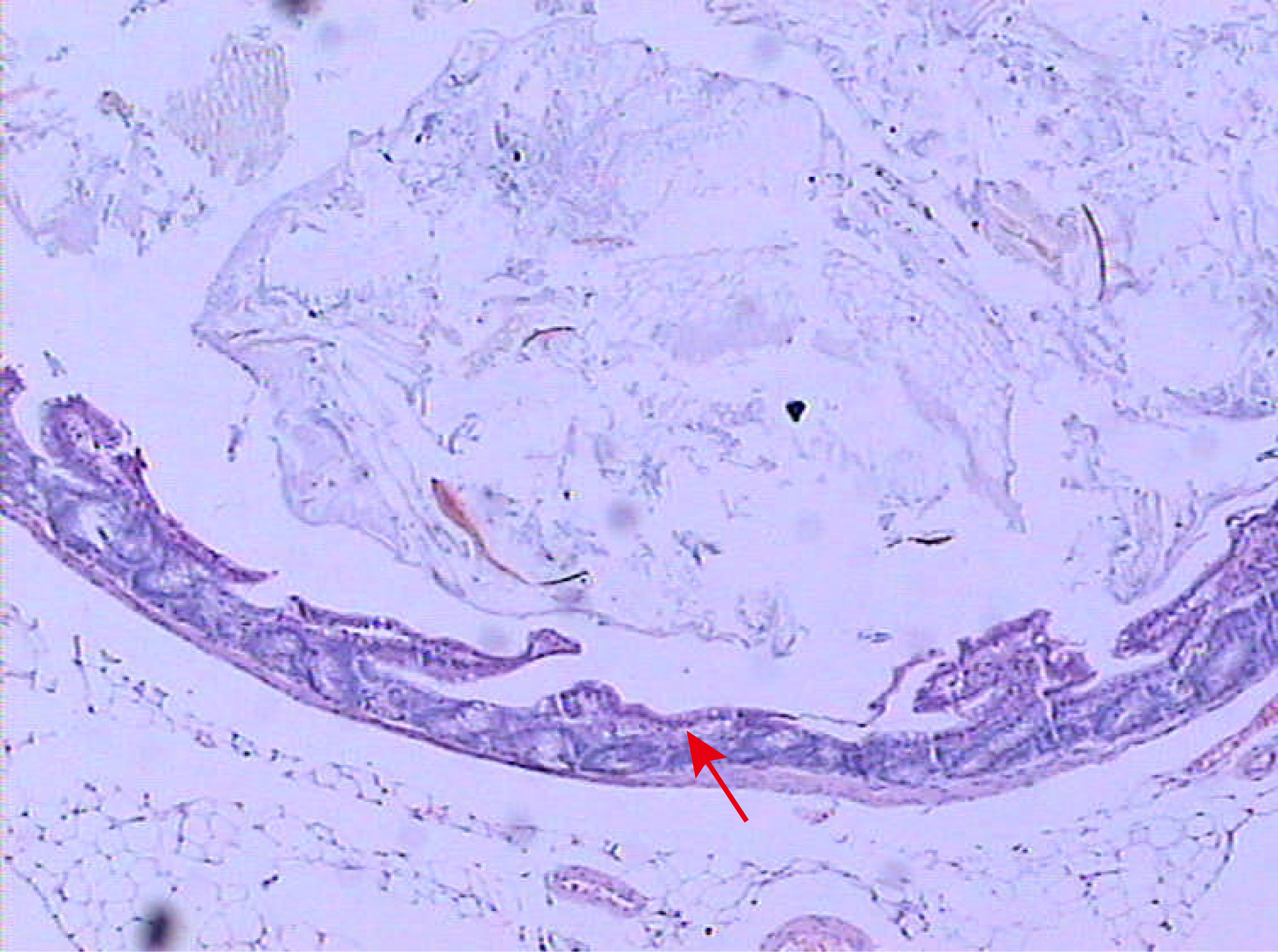

Supplement: Supplementary file 3 [file Data_Sheet_3.ZIP › Small Intestine/D0.jpg]

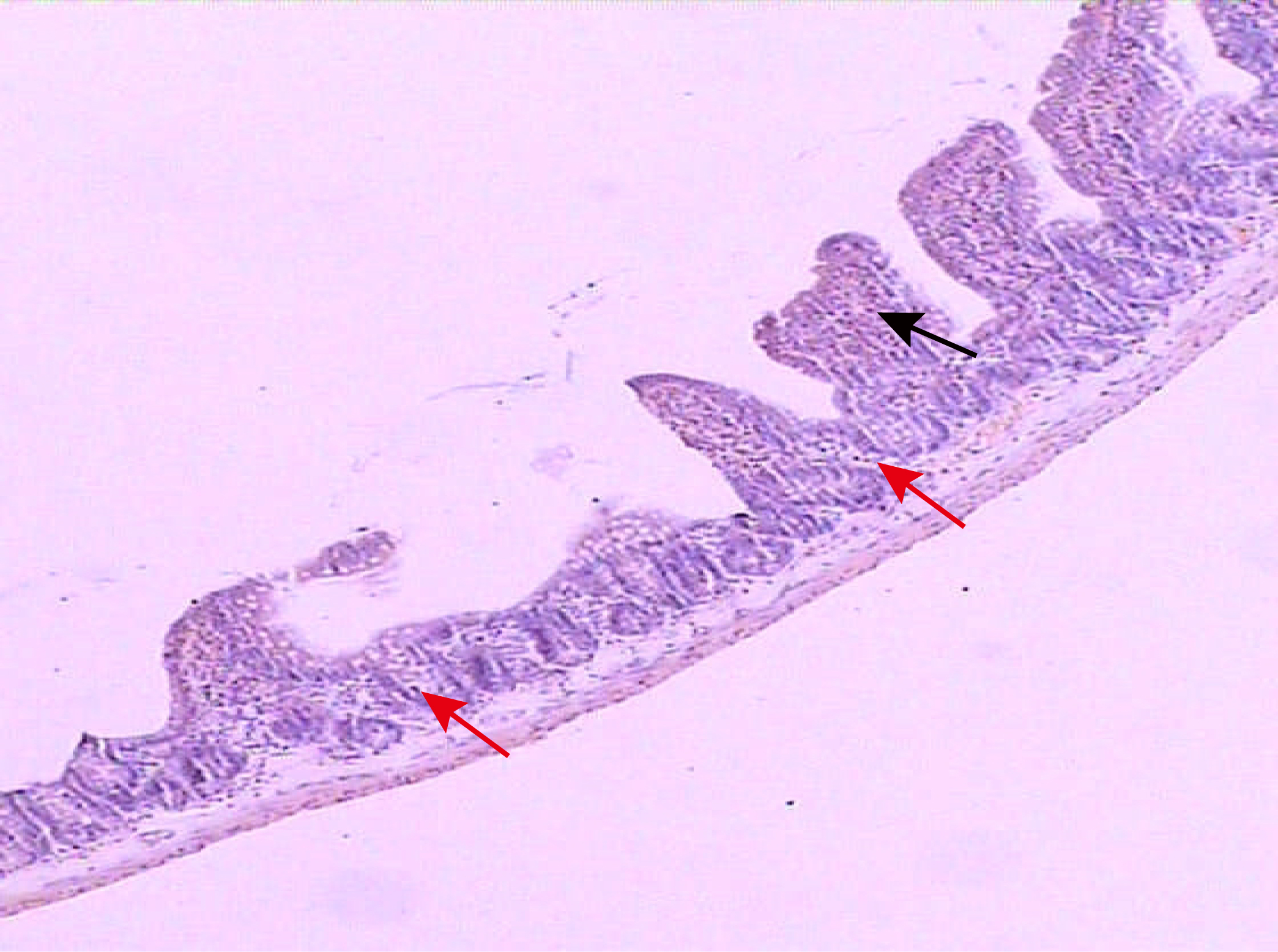

Supplement: Supplementary file 3 [file Data_Sheet_3.ZIP › Small Intestine/D24.jpg]

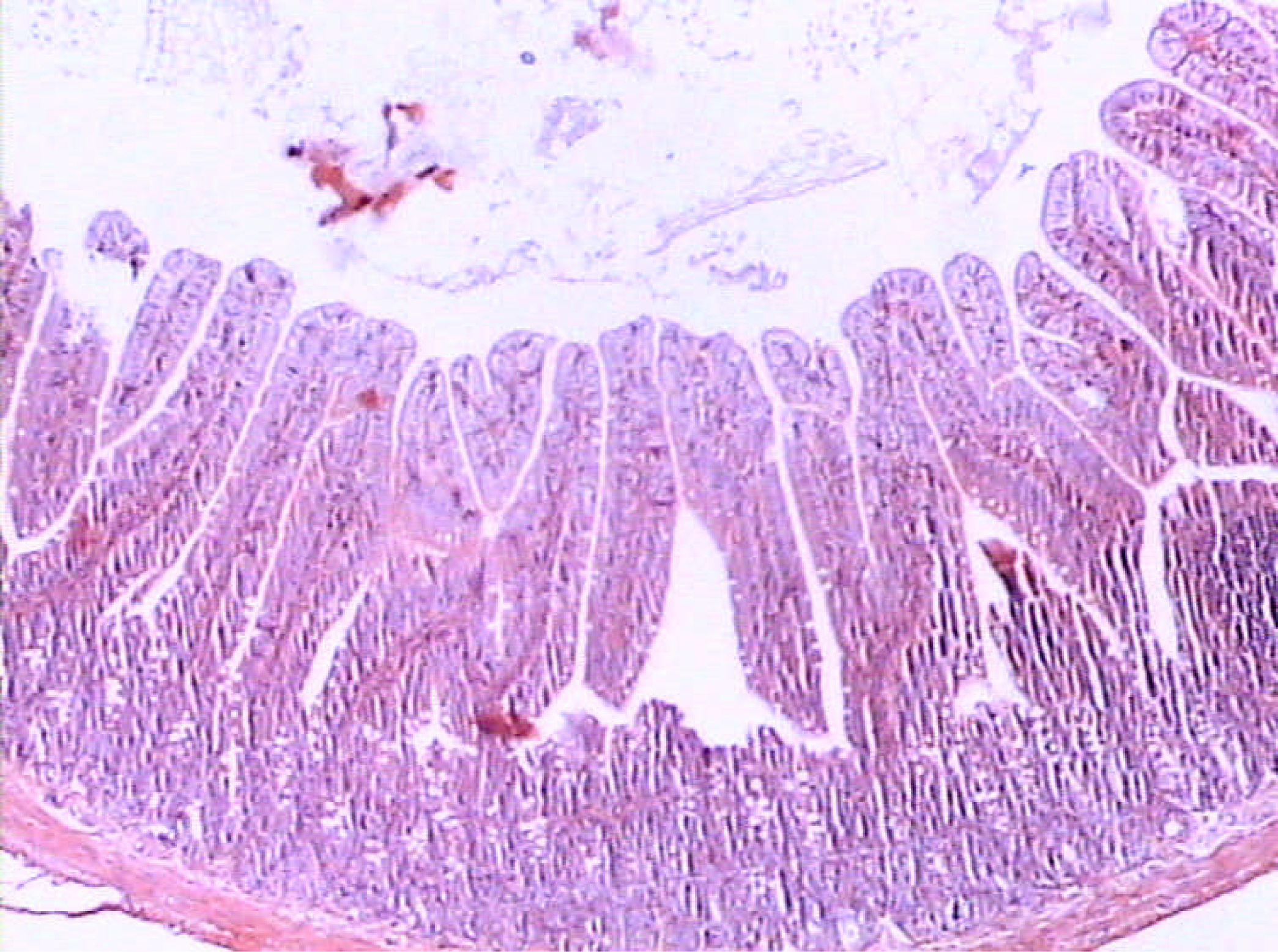

Supplement: Supplementary file 3 [file Data_Sheet_3.ZIP › Small Intestine/S.jpg]

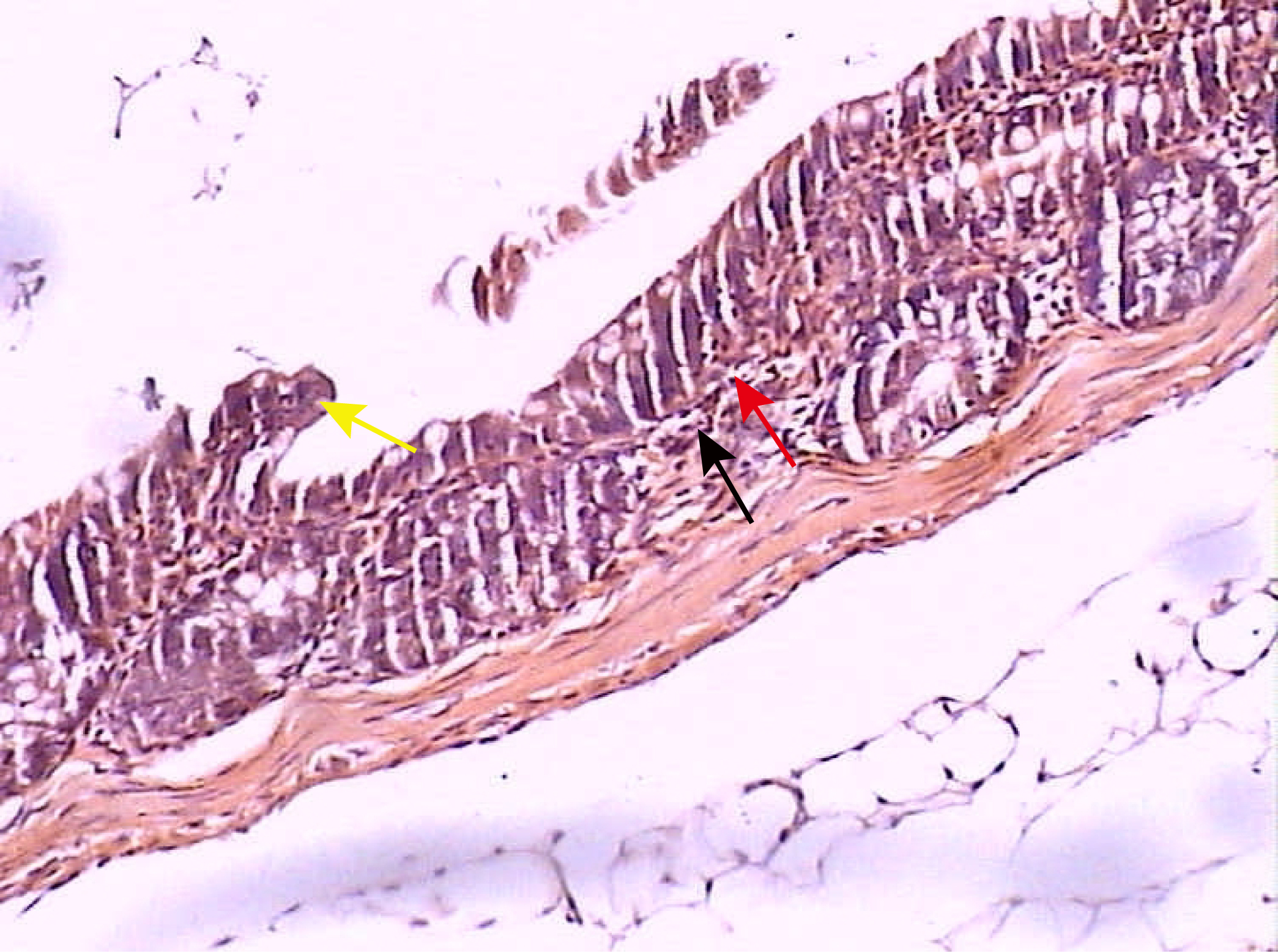

Supplement: Supplementary file 3 [file Data_Sheet_3.ZIP › Small Intestine/U0.jpg]

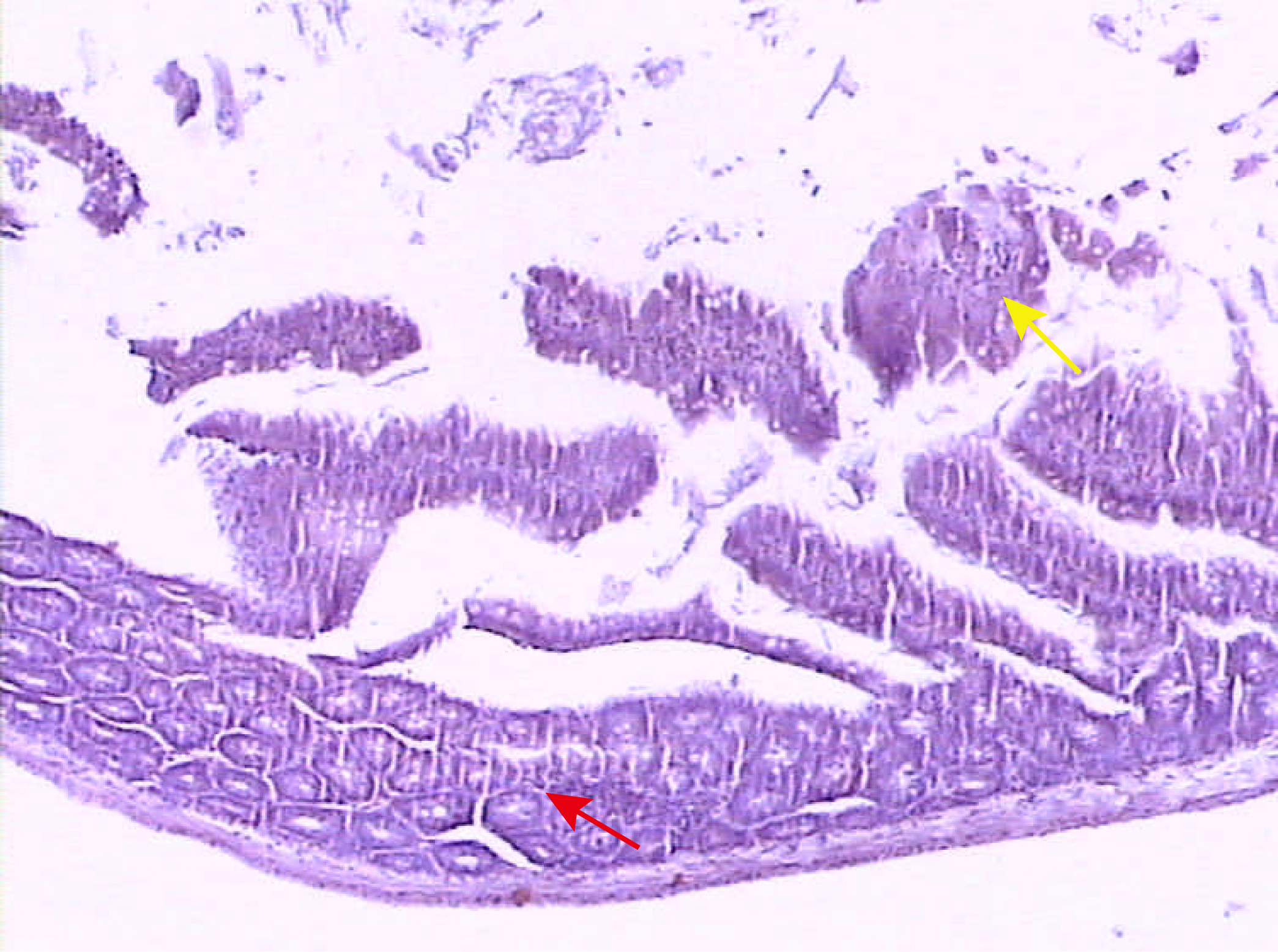

Supplement: Supplementary file 3 [file Data_Sheet_3.ZIP › Small Intestine/U24.jpg]

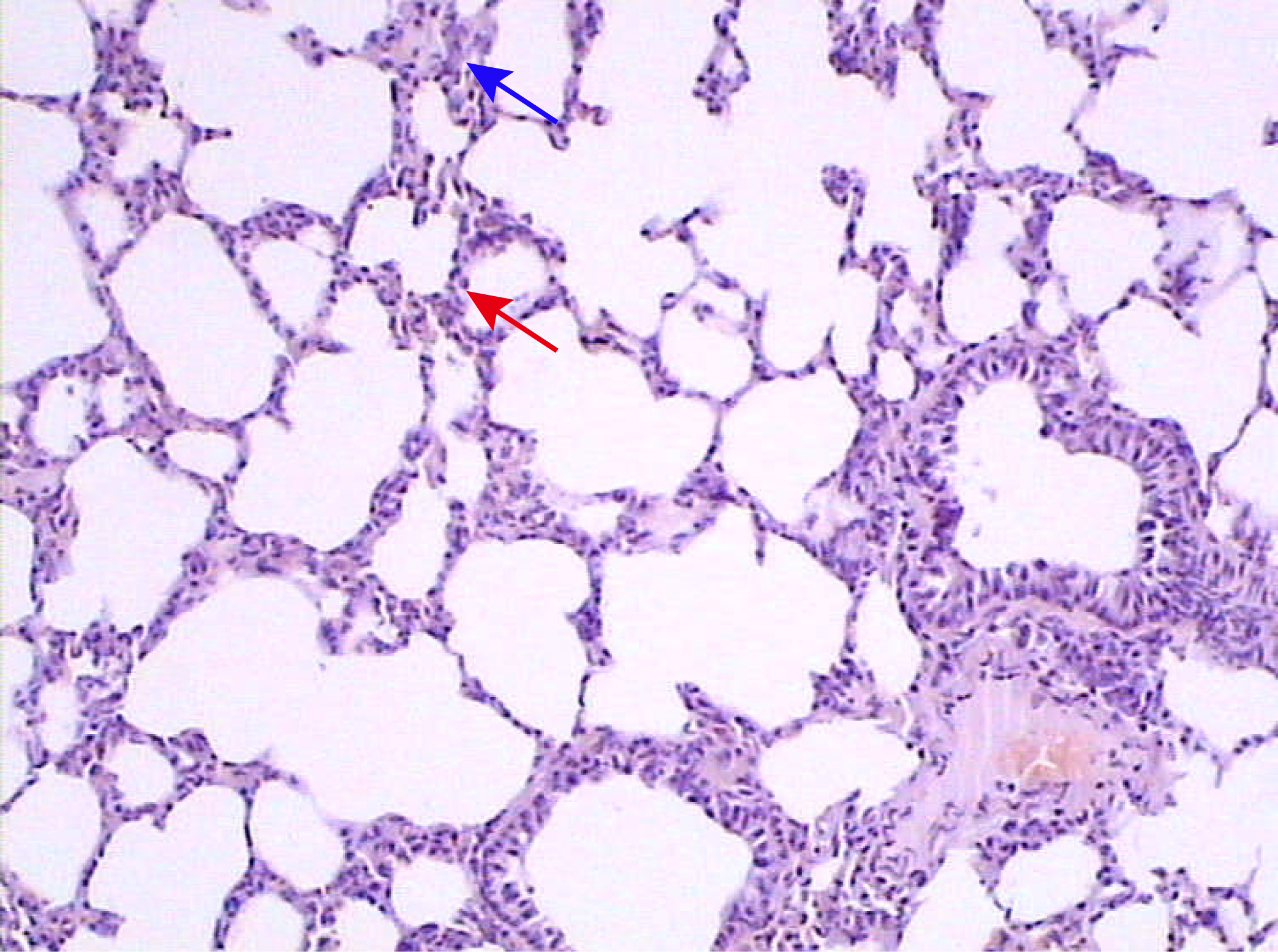

Supplement: Supplementary file 4 [file Data_Sheet_4.ZIP › Lung/D0.jpg]

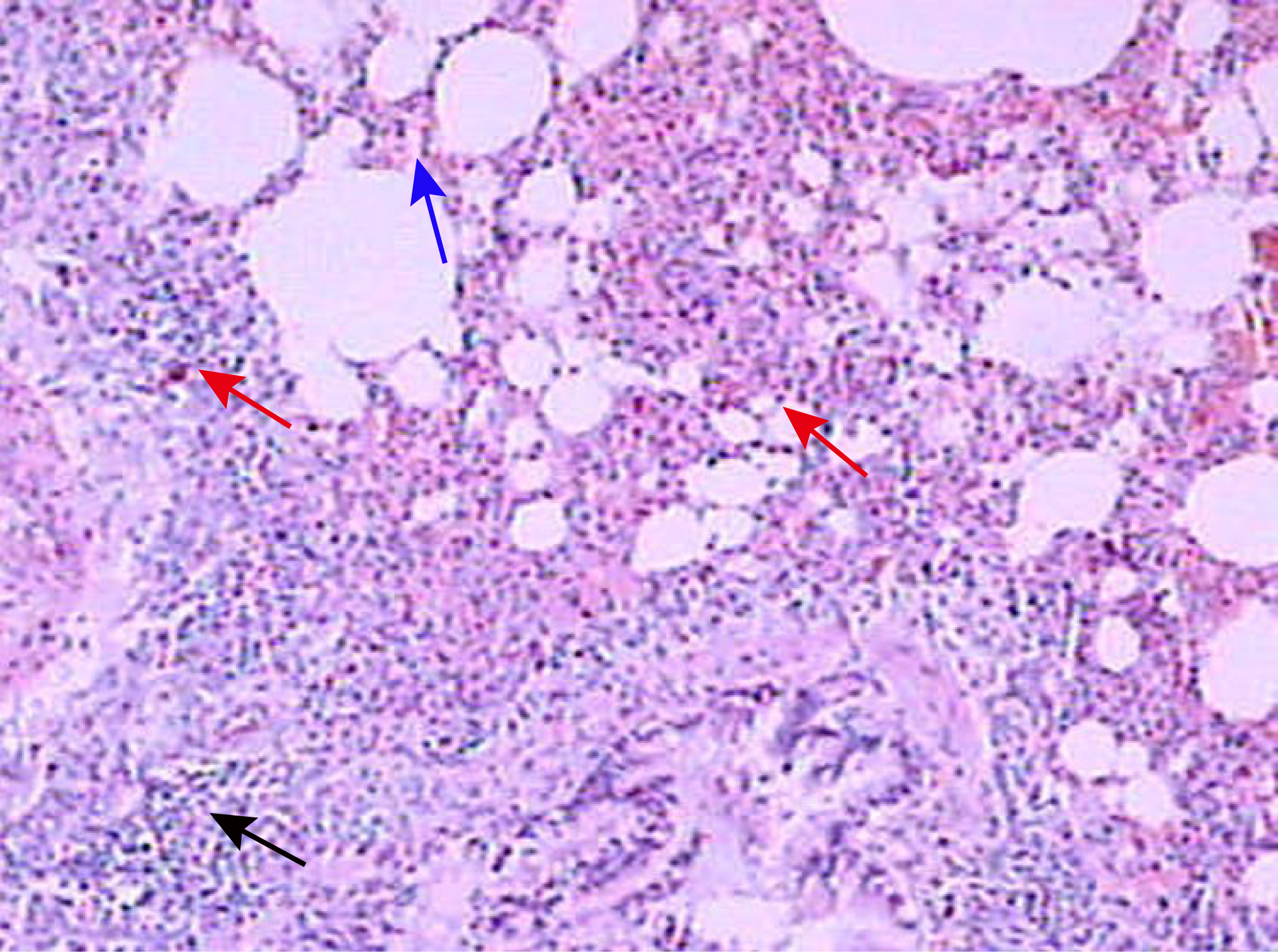

Supplement: Supplementary file 4 [file Data_Sheet_4.ZIP › Lung/D24.jpg]

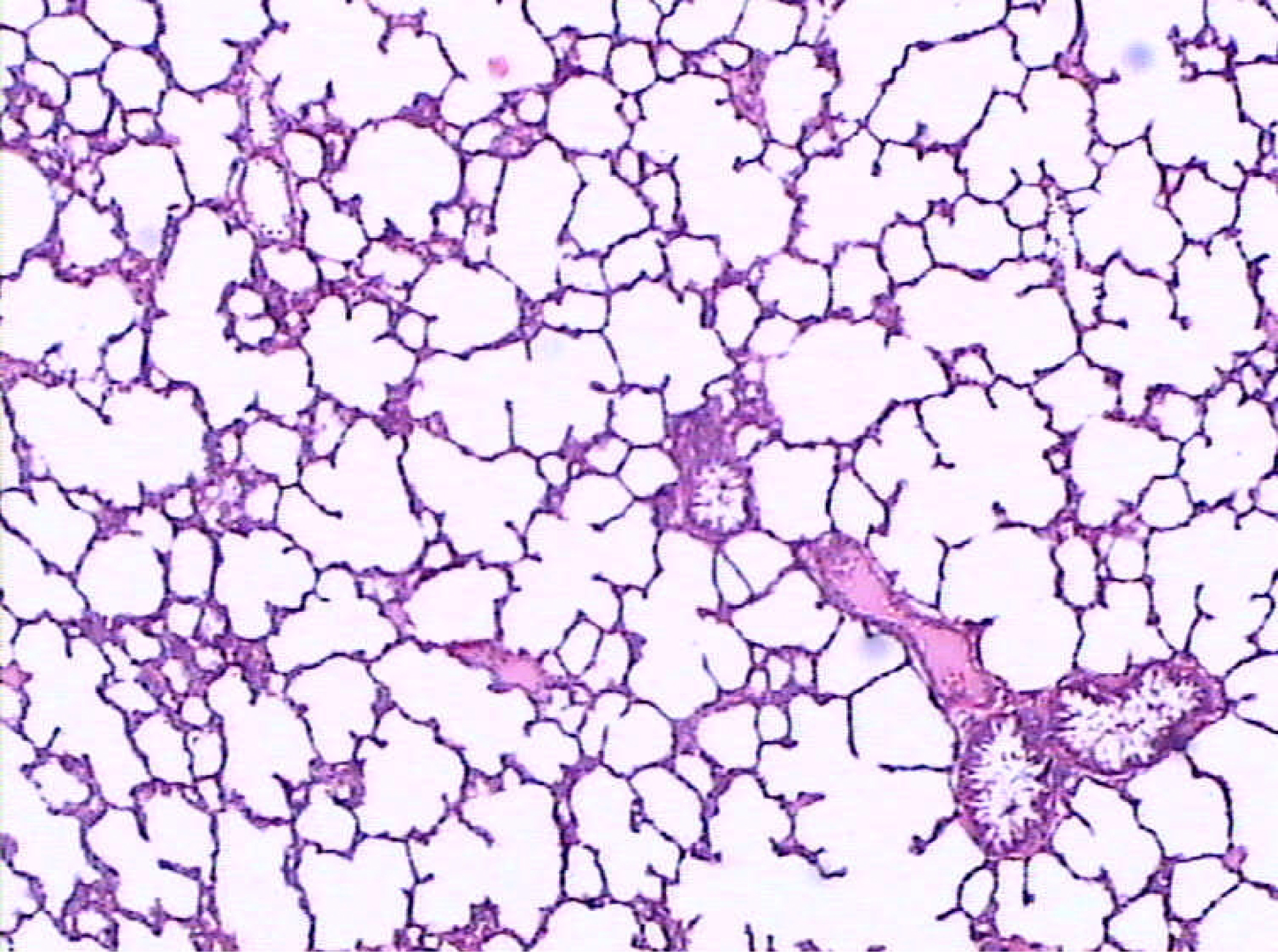

Supplement: Supplementary file 4 [file Data_Sheet_4.ZIP › Lung/S.jpg]

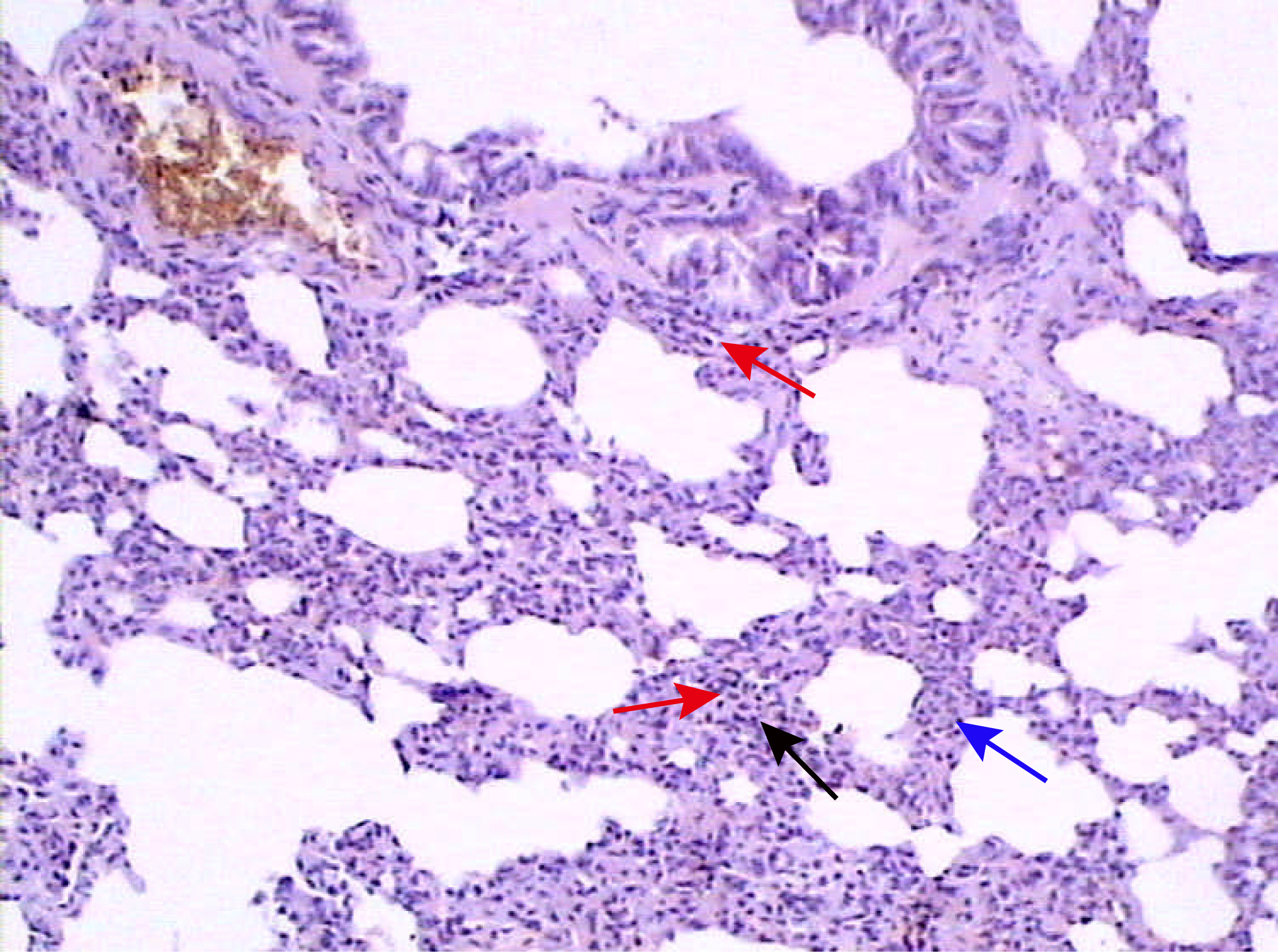

Supplement: Supplementary file 4 [file Data_Sheet_4.ZIP › Lung/U0.jpg]

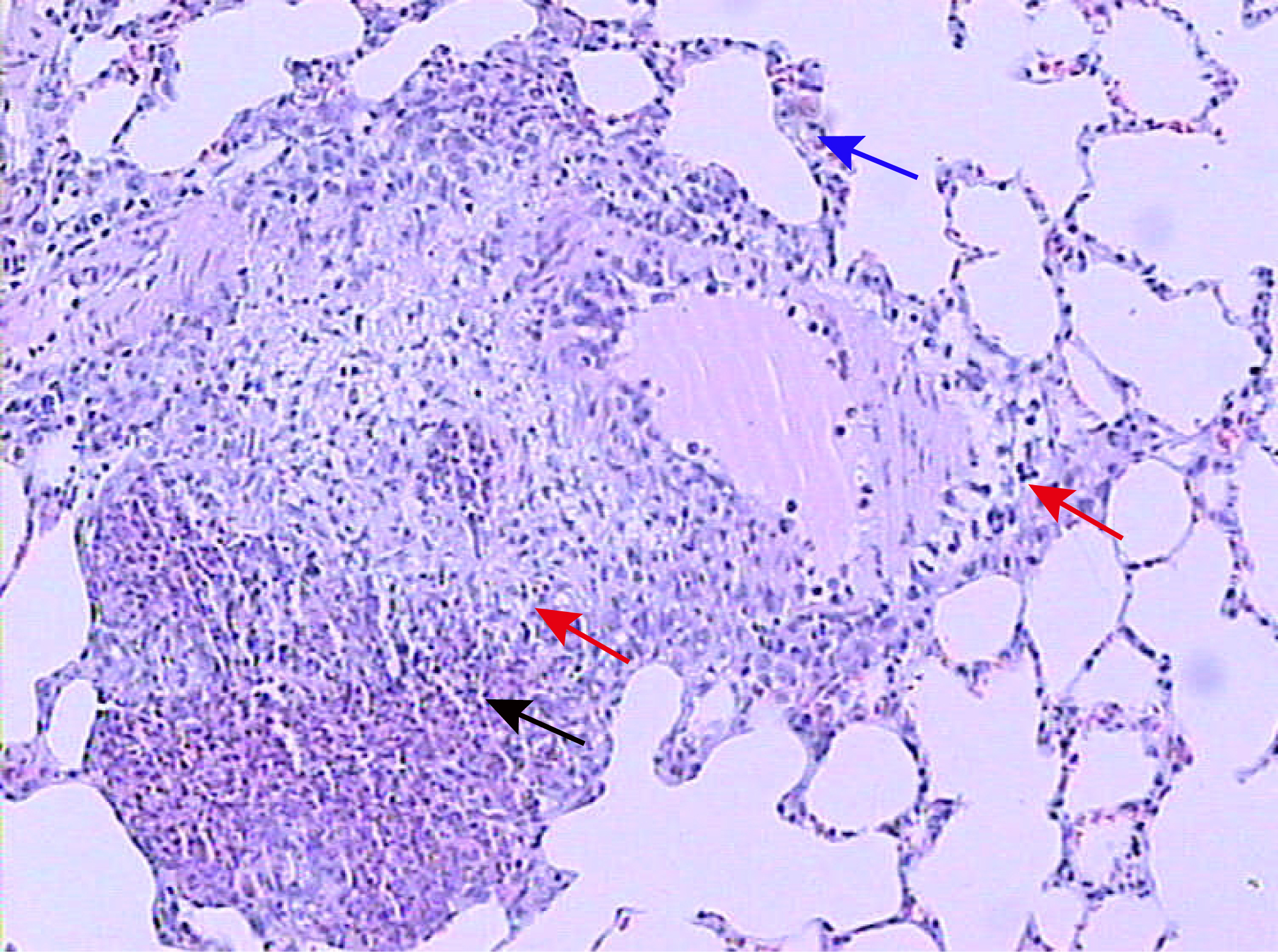

Supplement: Supplementary file 4 [file Data_Sheet_4.ZIP › Lung/U24.jpg]

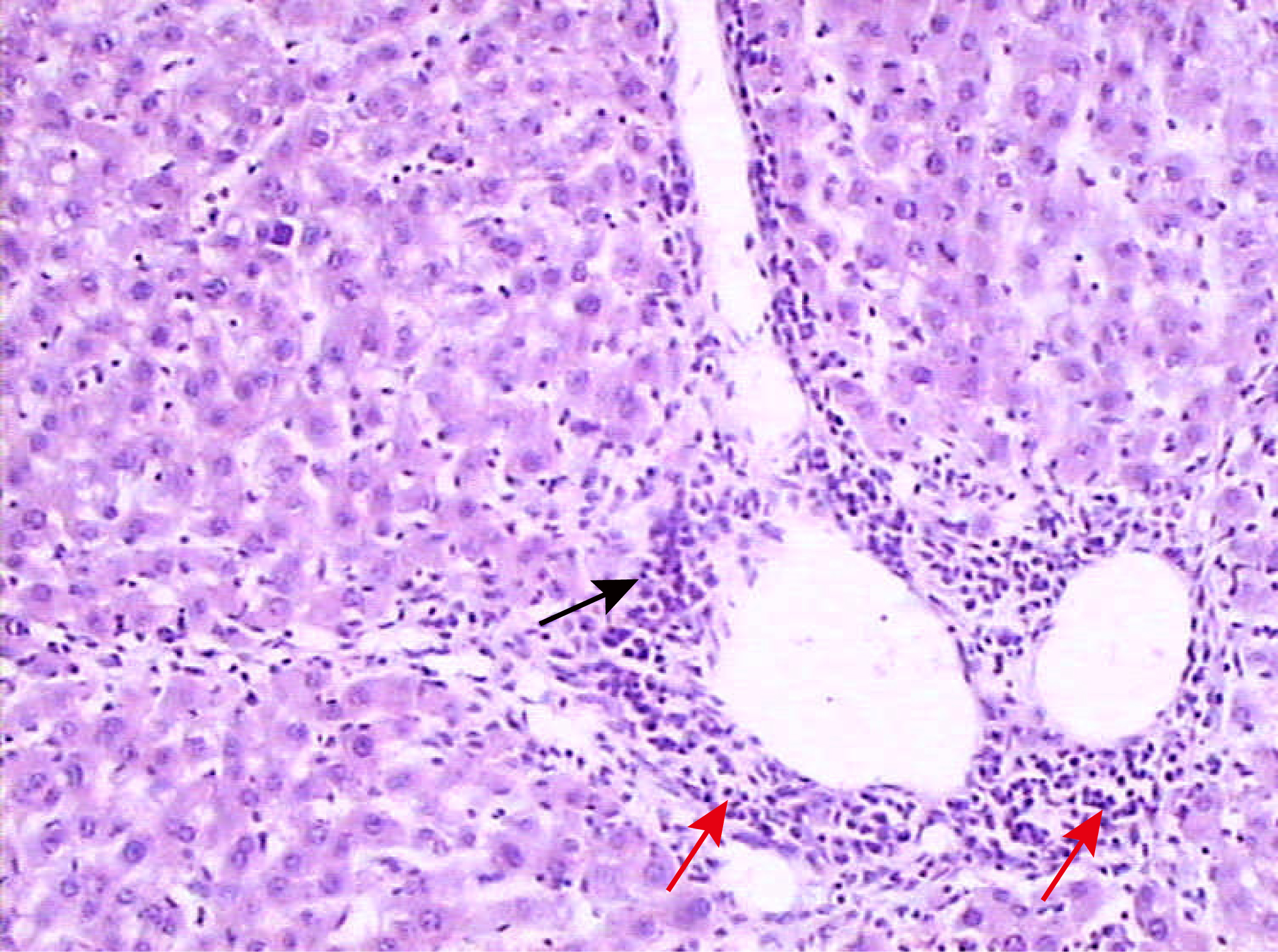

Supplement: Supplementary file 5 [file Data_Sheet_5.ZIP › Liver/D0.jpg]

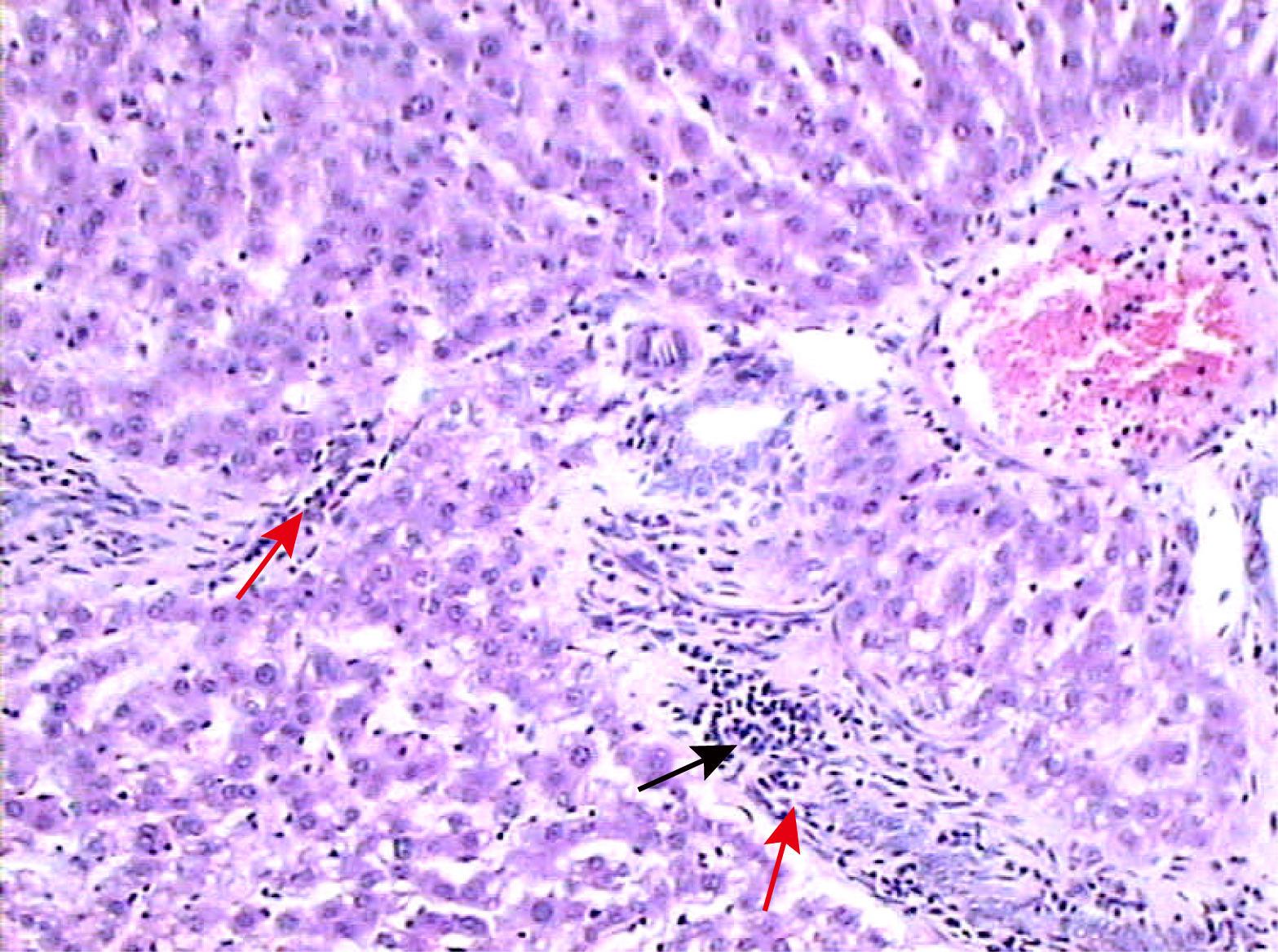

Supplement: Supplementary file 5 [file Data_Sheet_5.ZIP › Liver/D24.jpg]

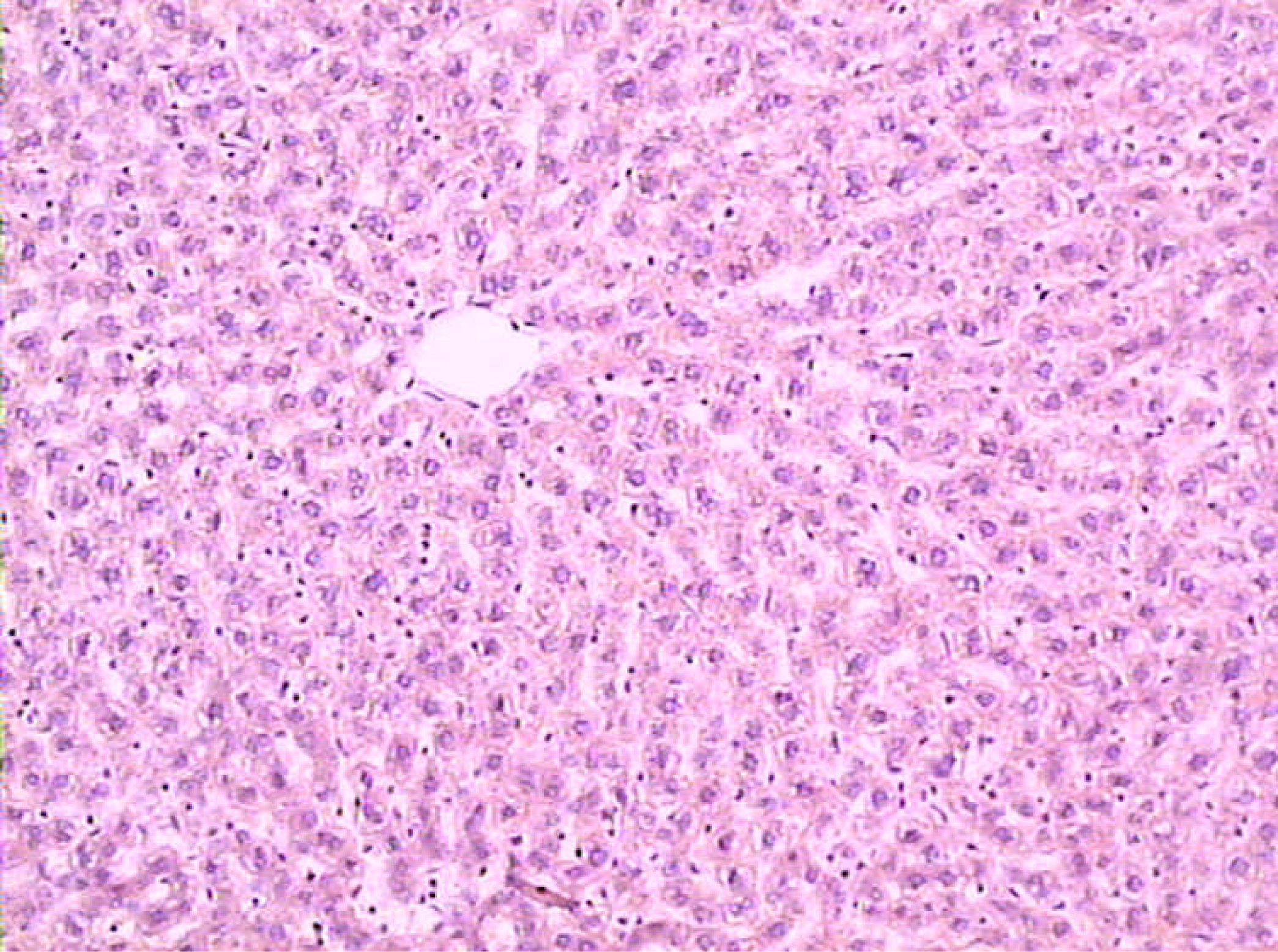

Supplement: Supplementary file 5 [file Data_Sheet_5.ZIP › Liver/S.jpg]

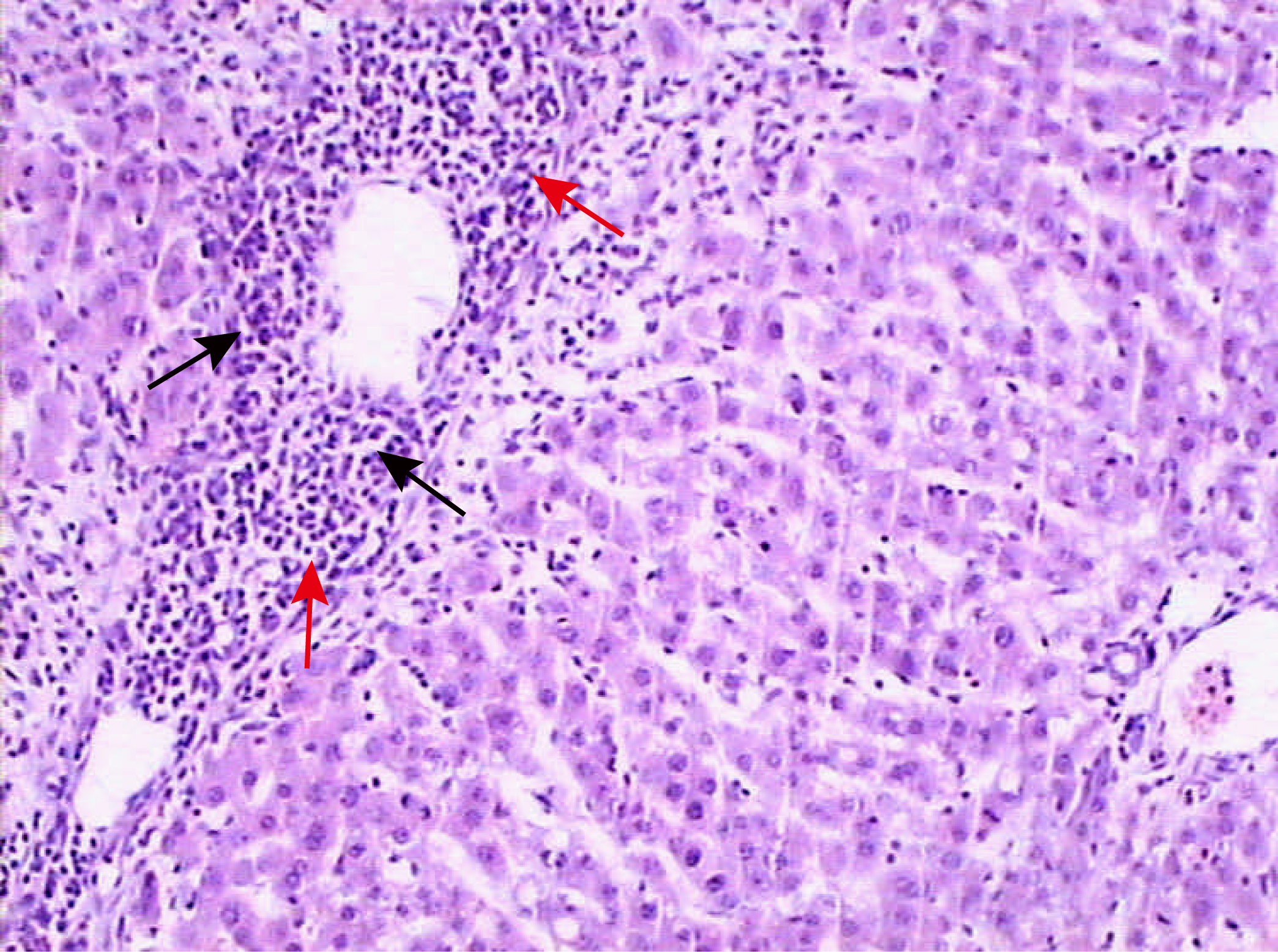

Supplement: Supplementary file 5 [file Data_Sheet_5.ZIP › Liver/U0.jpg]

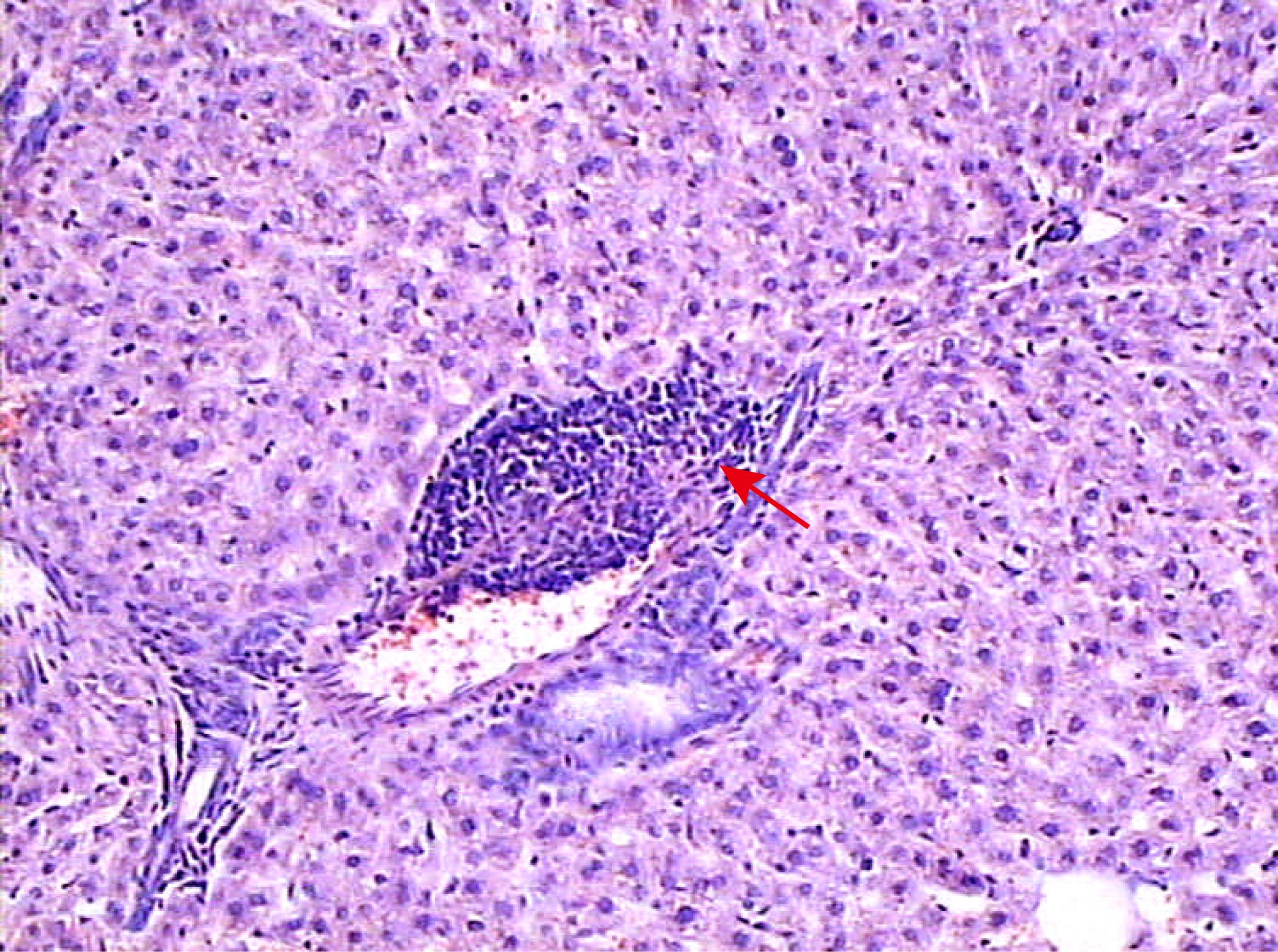

Supplement: Supplementary file 5 [file Data_Sheet_5.ZIP › Liver/U24.jpg]

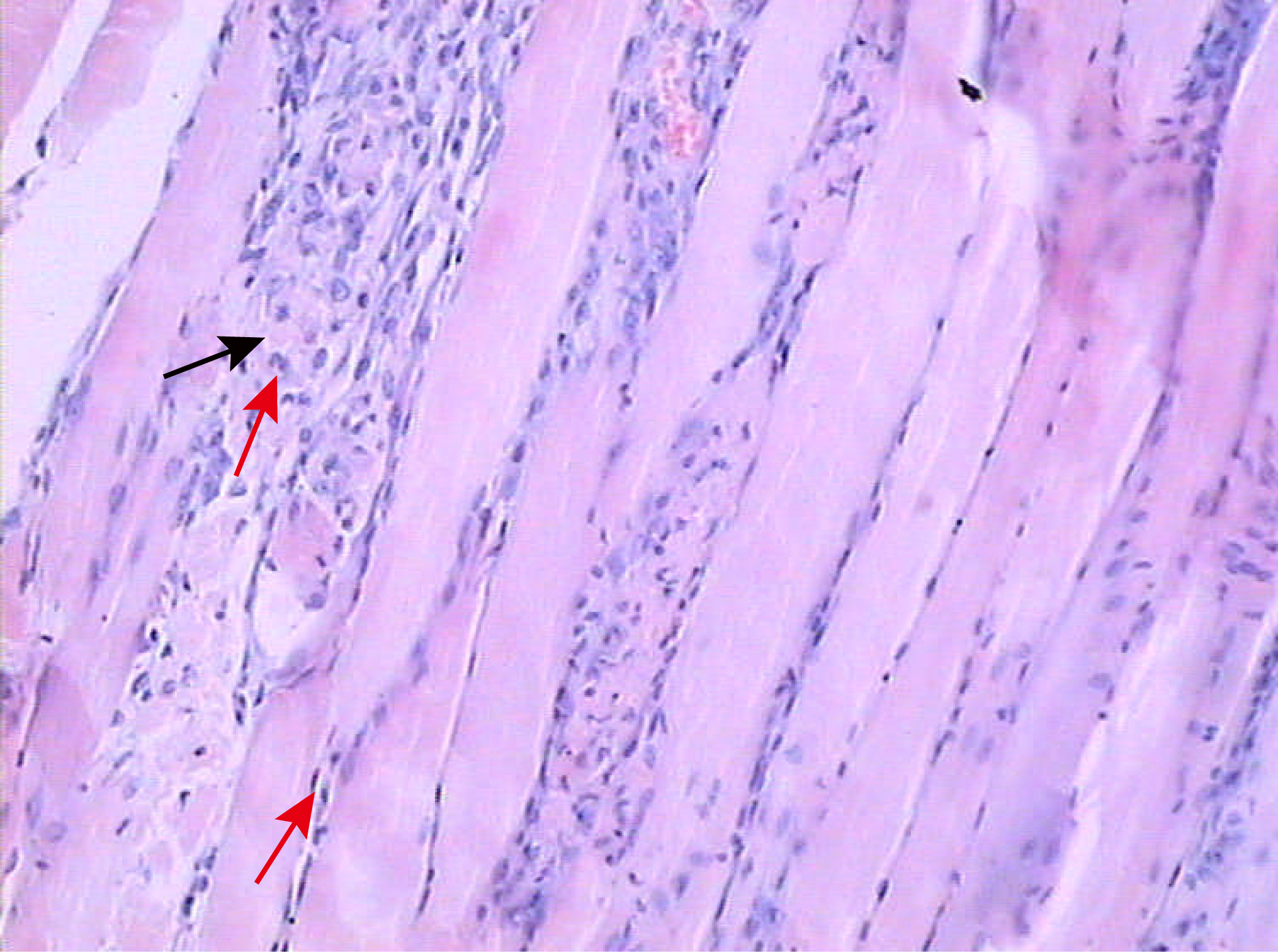

Supplement: Supplementary file 6 [file Data_Sheet_6.ZIP › Lateral Femoral Muscle/D0.jpg]

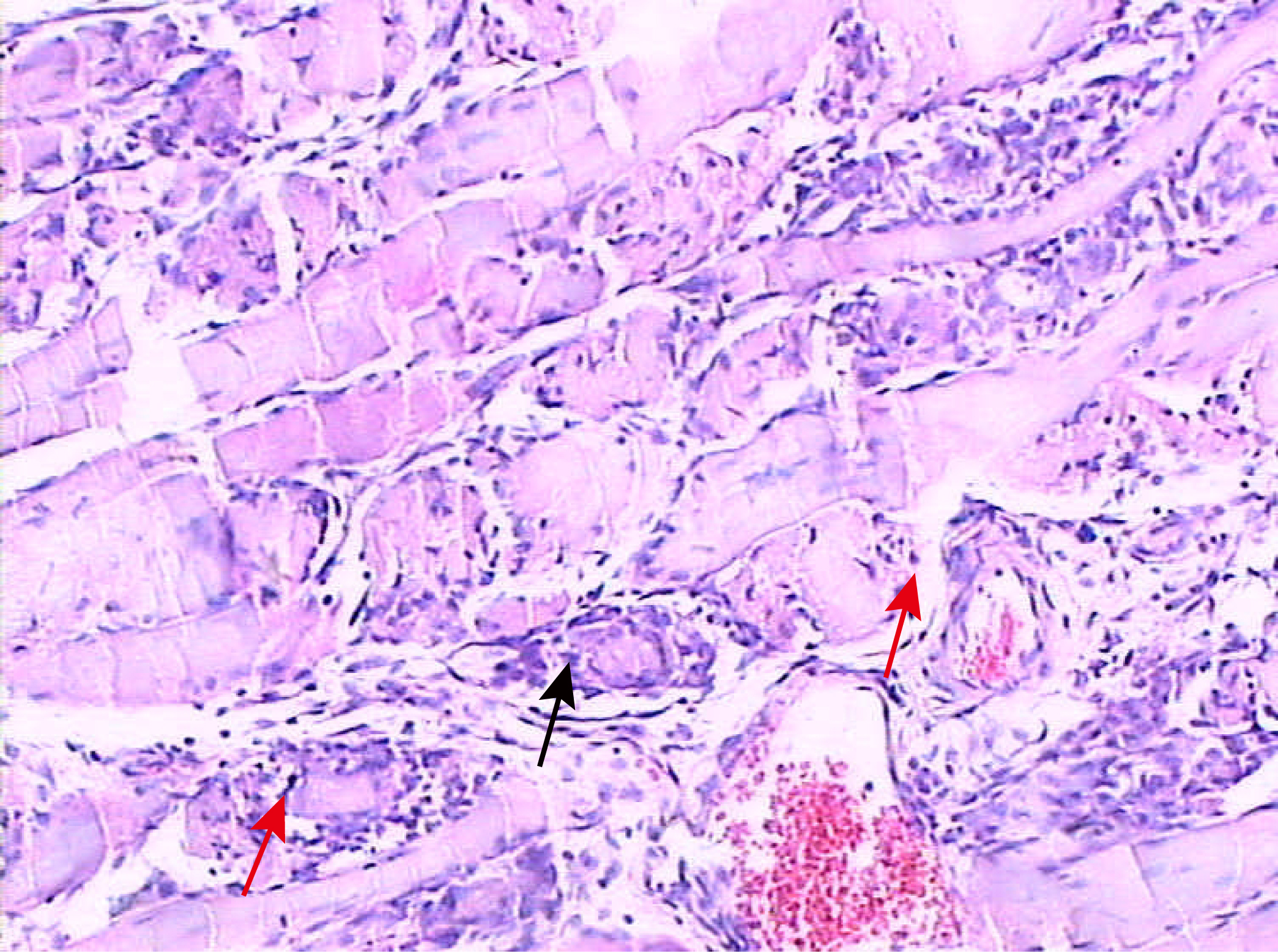

Supplement: Supplementary file 6 [file Data_Sheet_6.ZIP › Lateral Femoral Muscle/D24.jpg]

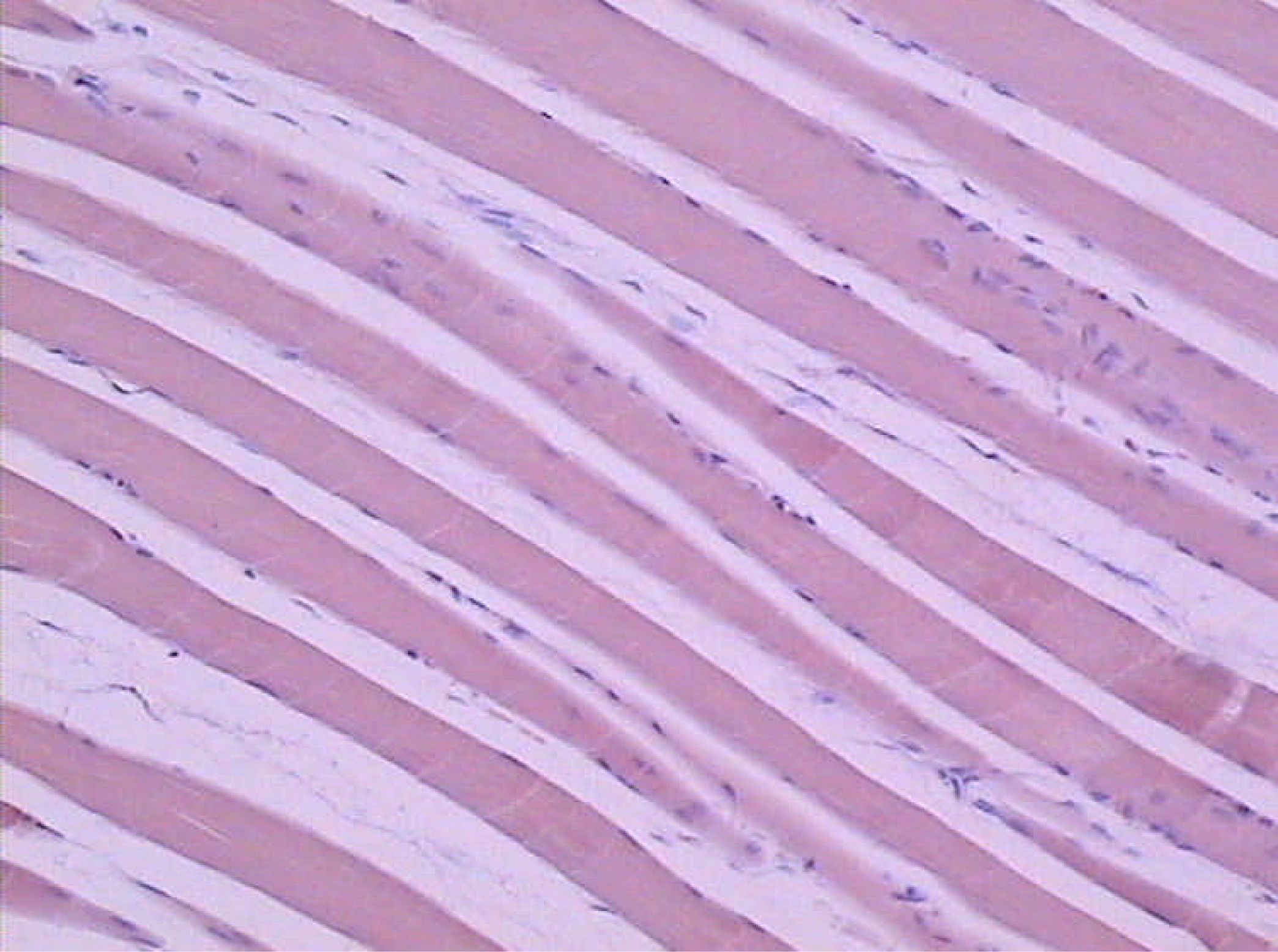

Supplement: Supplementary file 6 [file Data_Sheet_6.ZIP › Lateral Femoral Muscle/S.jpg]

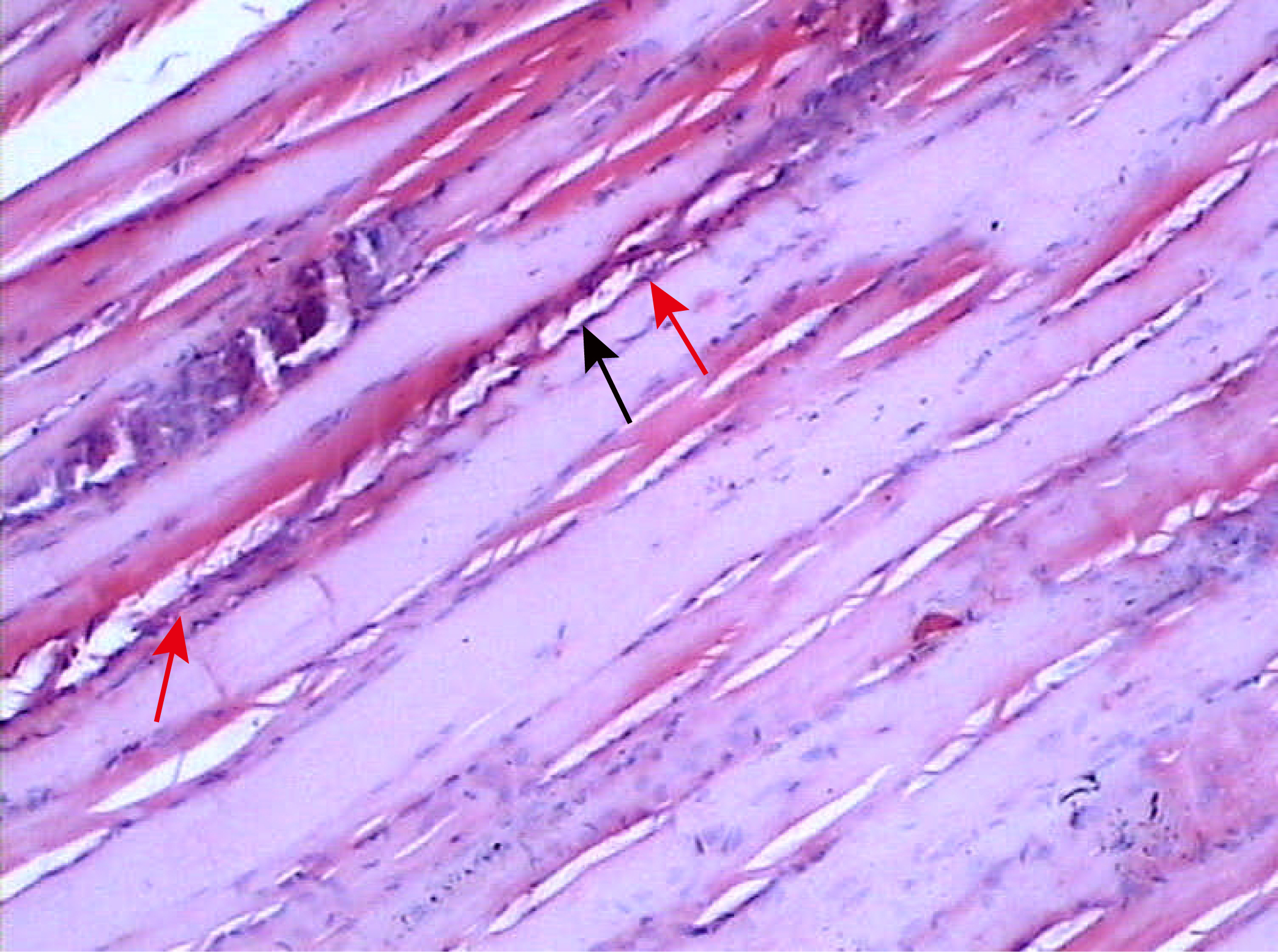

Supplement: Supplementary file 6 [file Data_Sheet_6.ZIP › Lateral Femoral Muscle/U0.jpg]

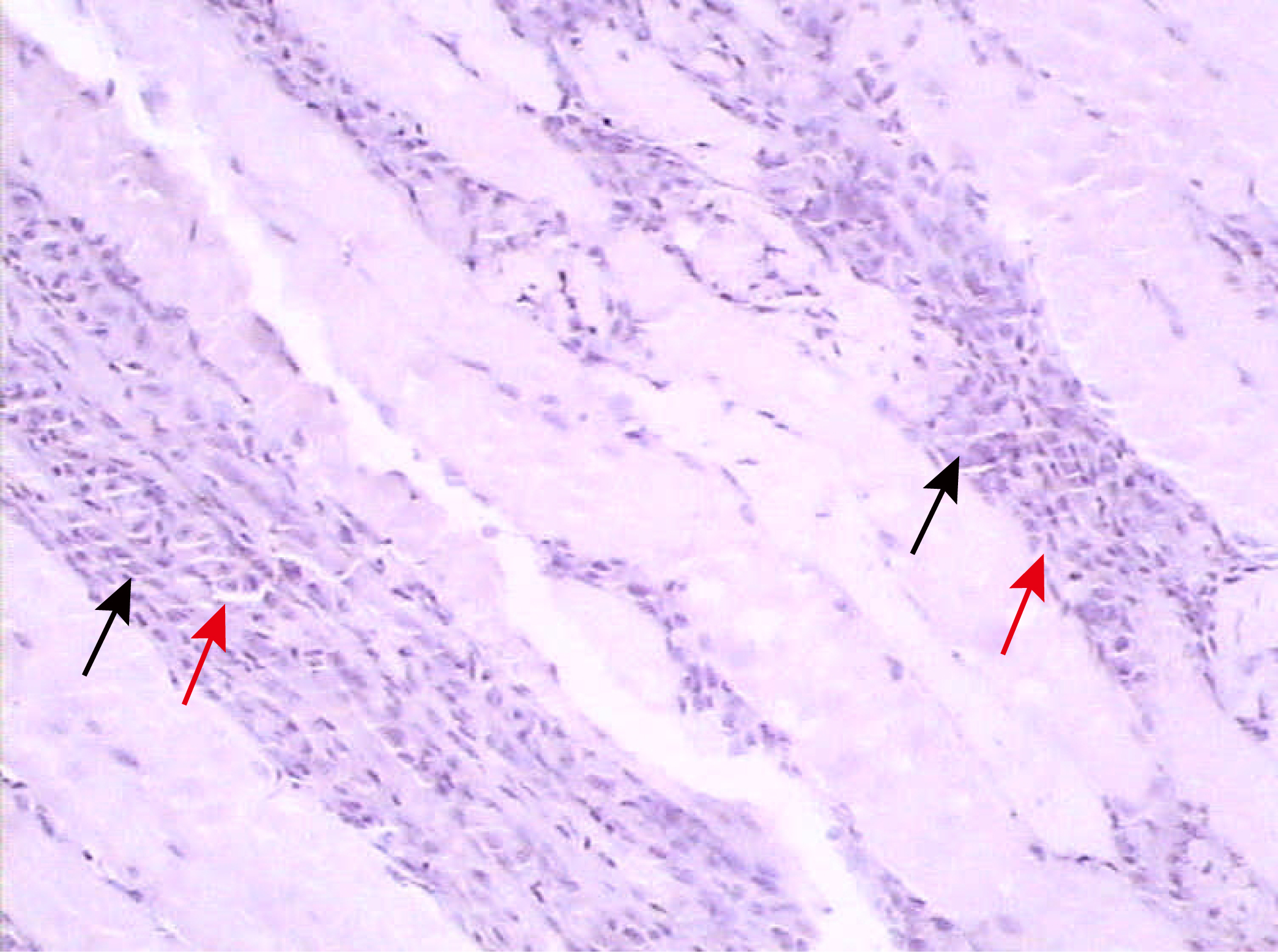

Supplement: Supplementary file 6 [file Data_Sheet_6.ZIP › Lateral Femoral Muscle/U24.jpg]

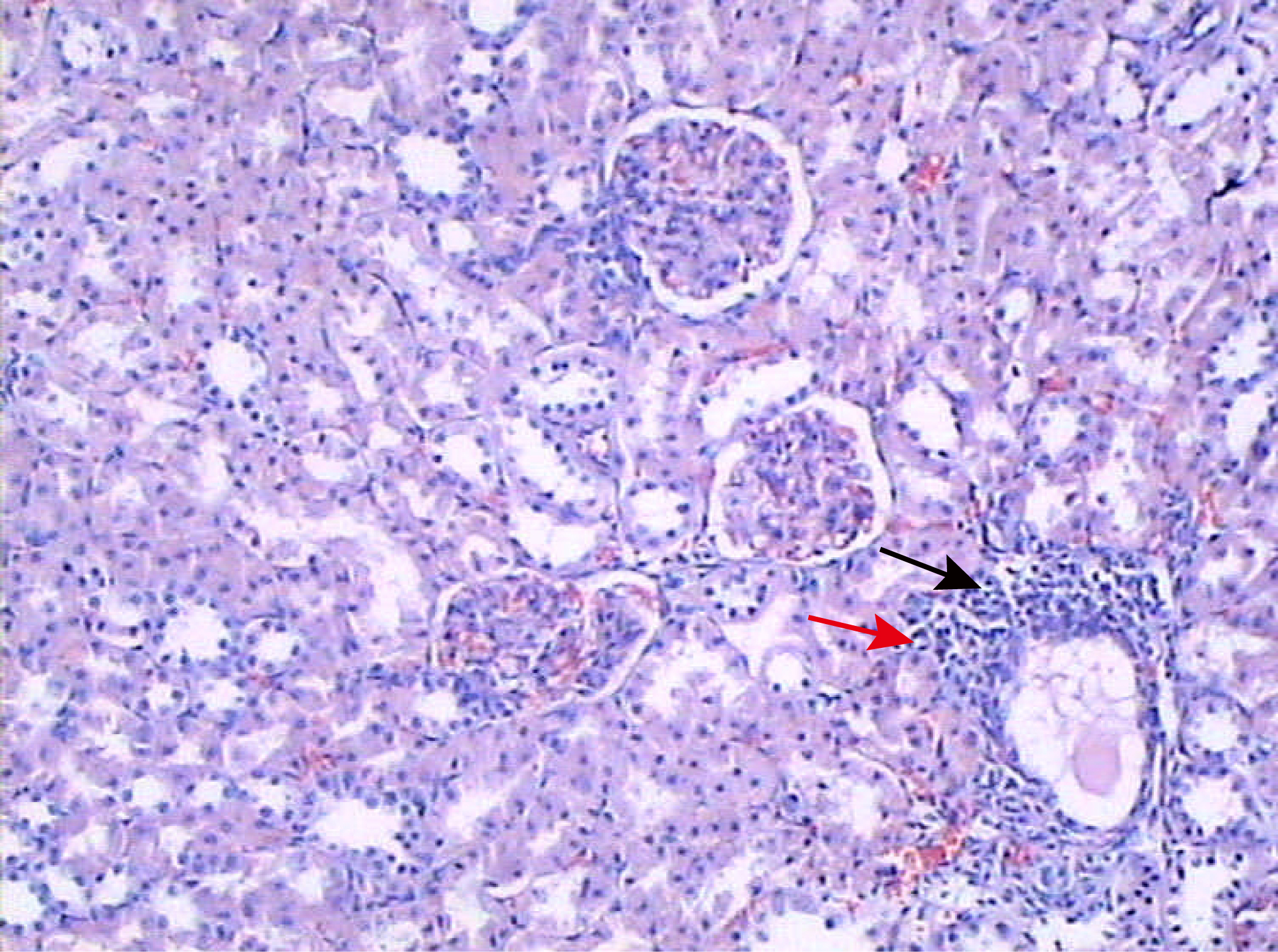

Supplement: Supplementary file 7 [file Data_Sheet_7.ZIP › Kidney/D0.jpg]

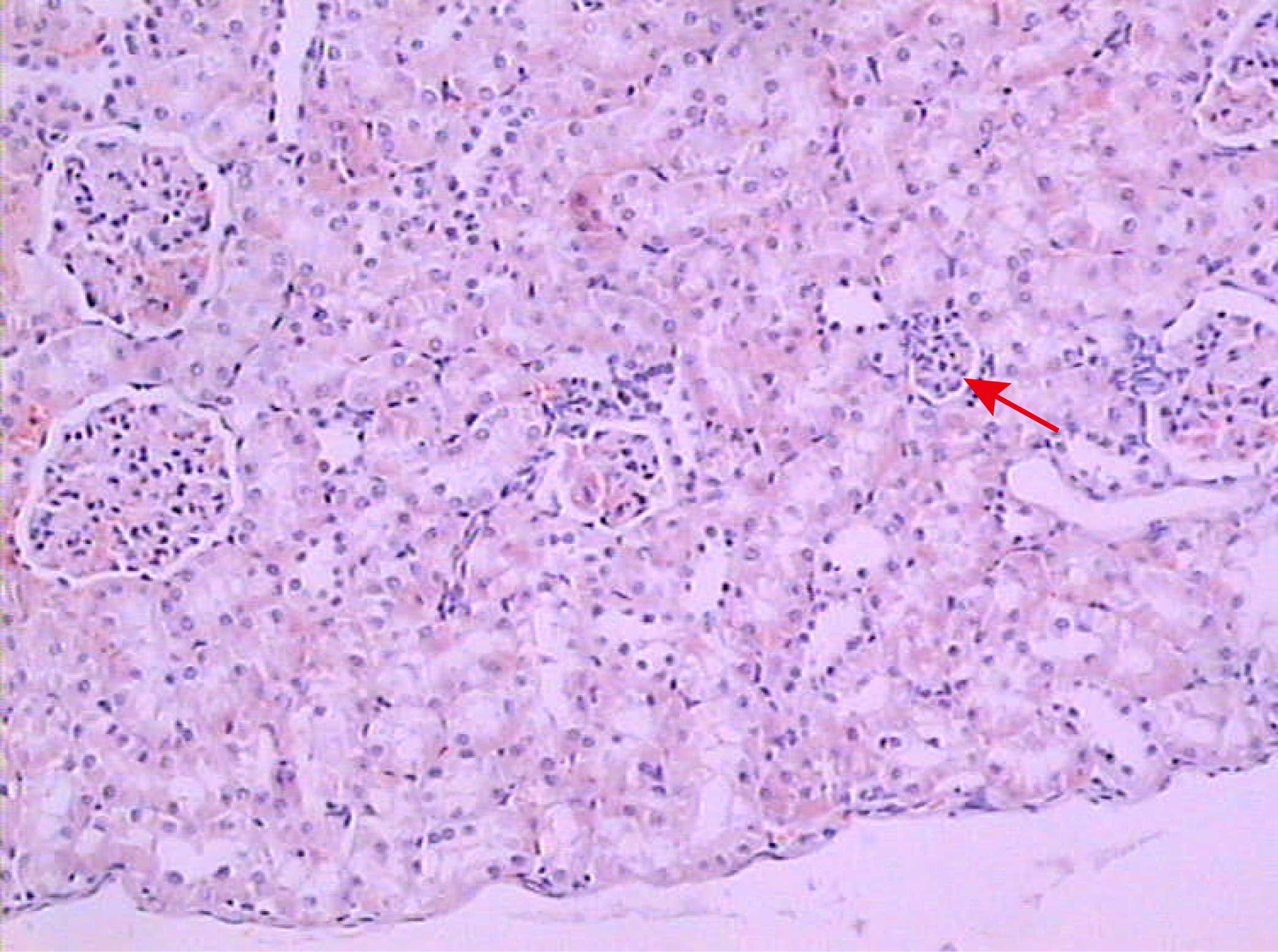

Supplement: Supplementary file 7 [file Data_Sheet_7.ZIP › Kidney/D24.jpg]

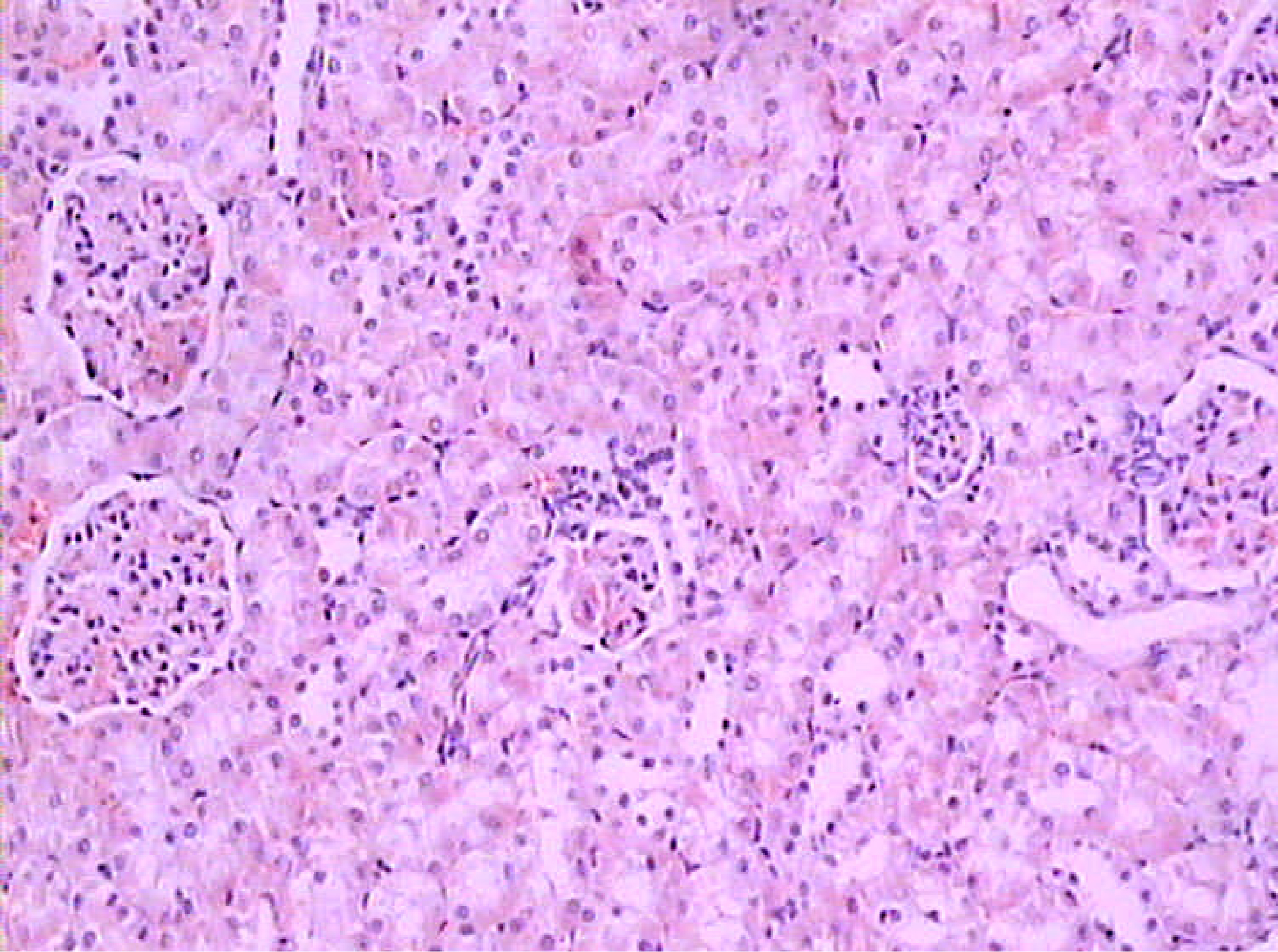

Supplement: Supplementary file 7 [file Data_Sheet_7.ZIP › Kidney/S.jpg]

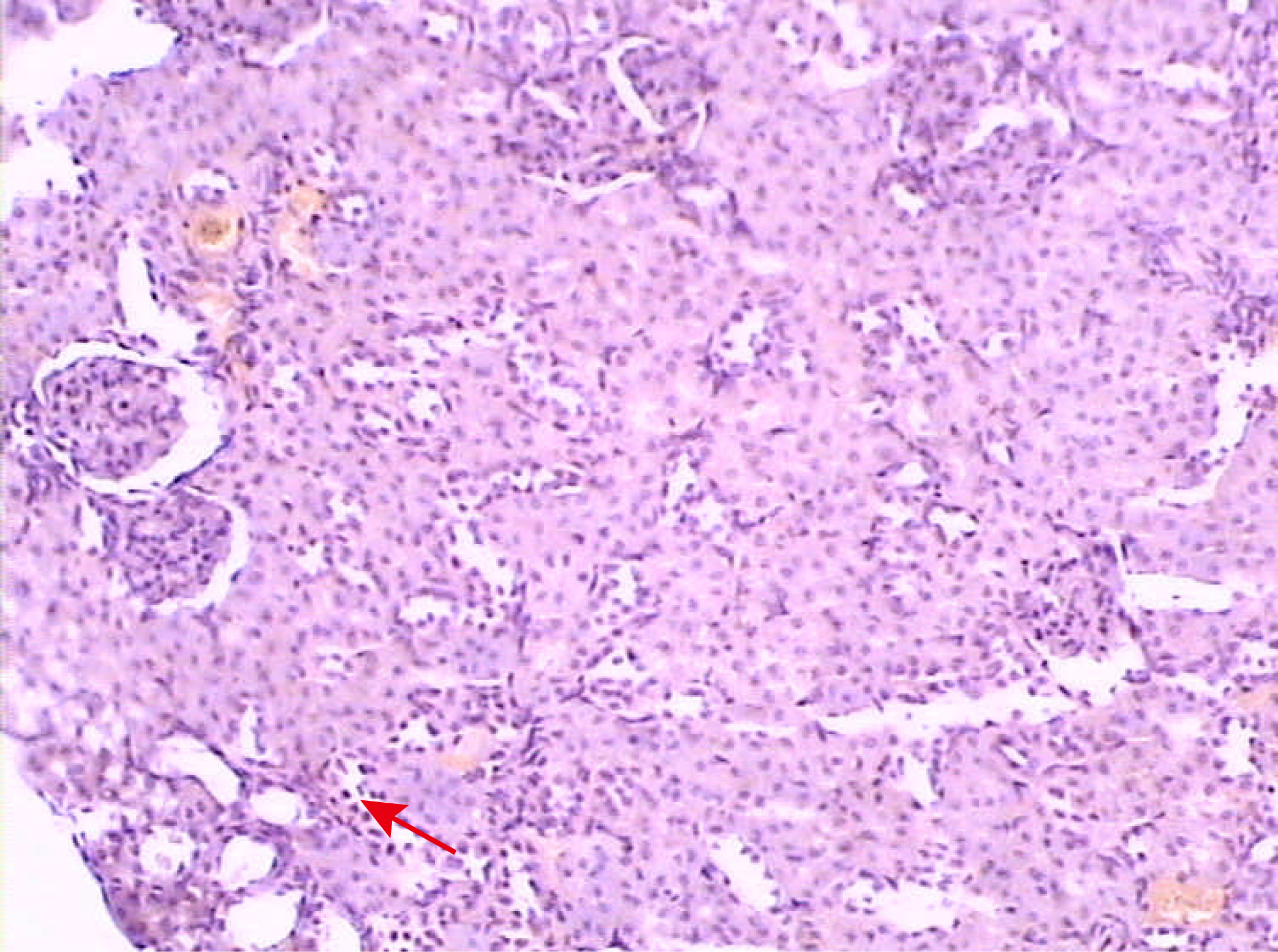

Supplement: Supplementary file 7 [file Data_Sheet_7.ZIP › Kidney/U0.jpg]

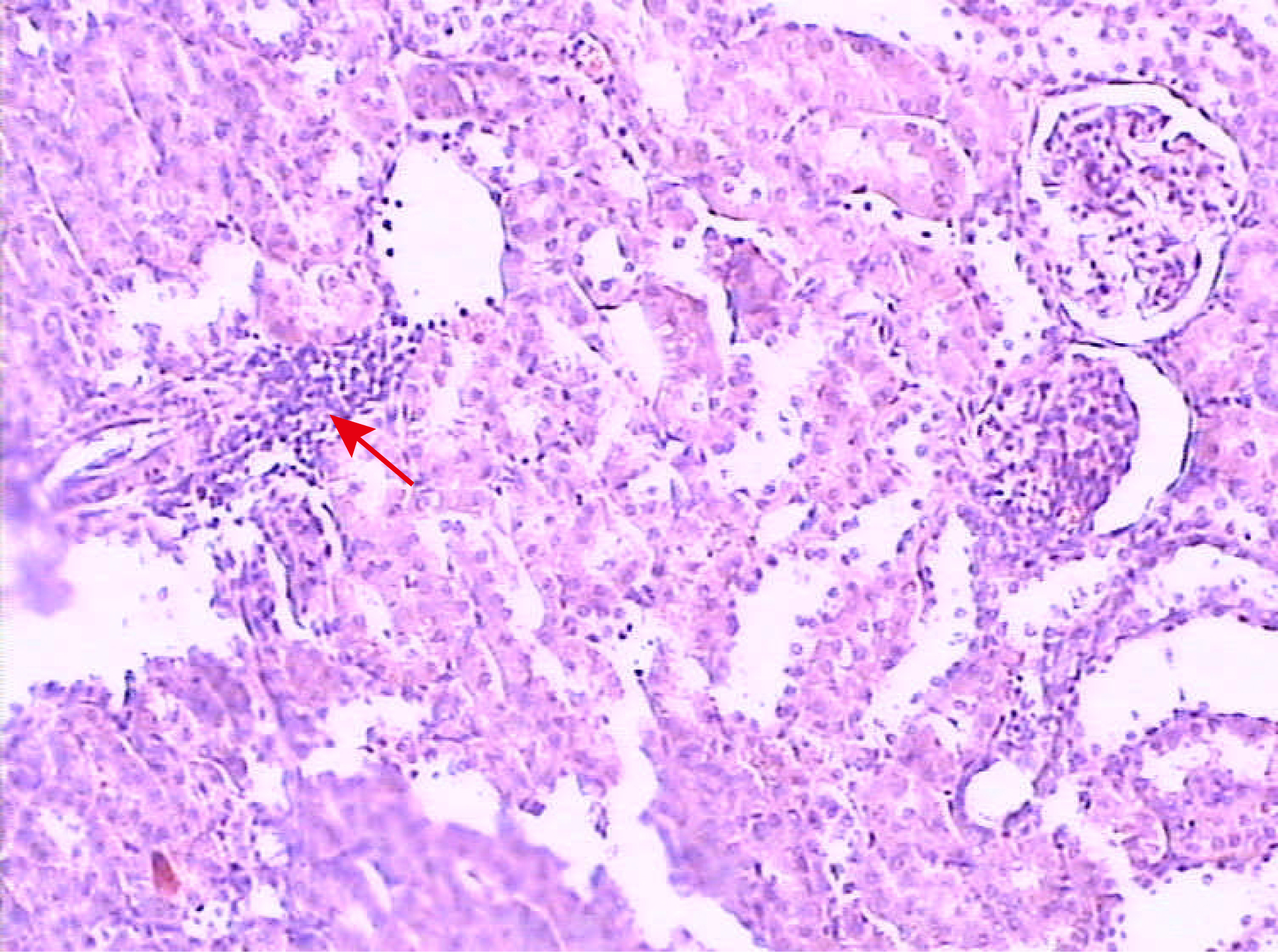

Supplement: Supplementary file 7 [file Data_Sheet_7.ZIP › Kidney/U24.jpg]

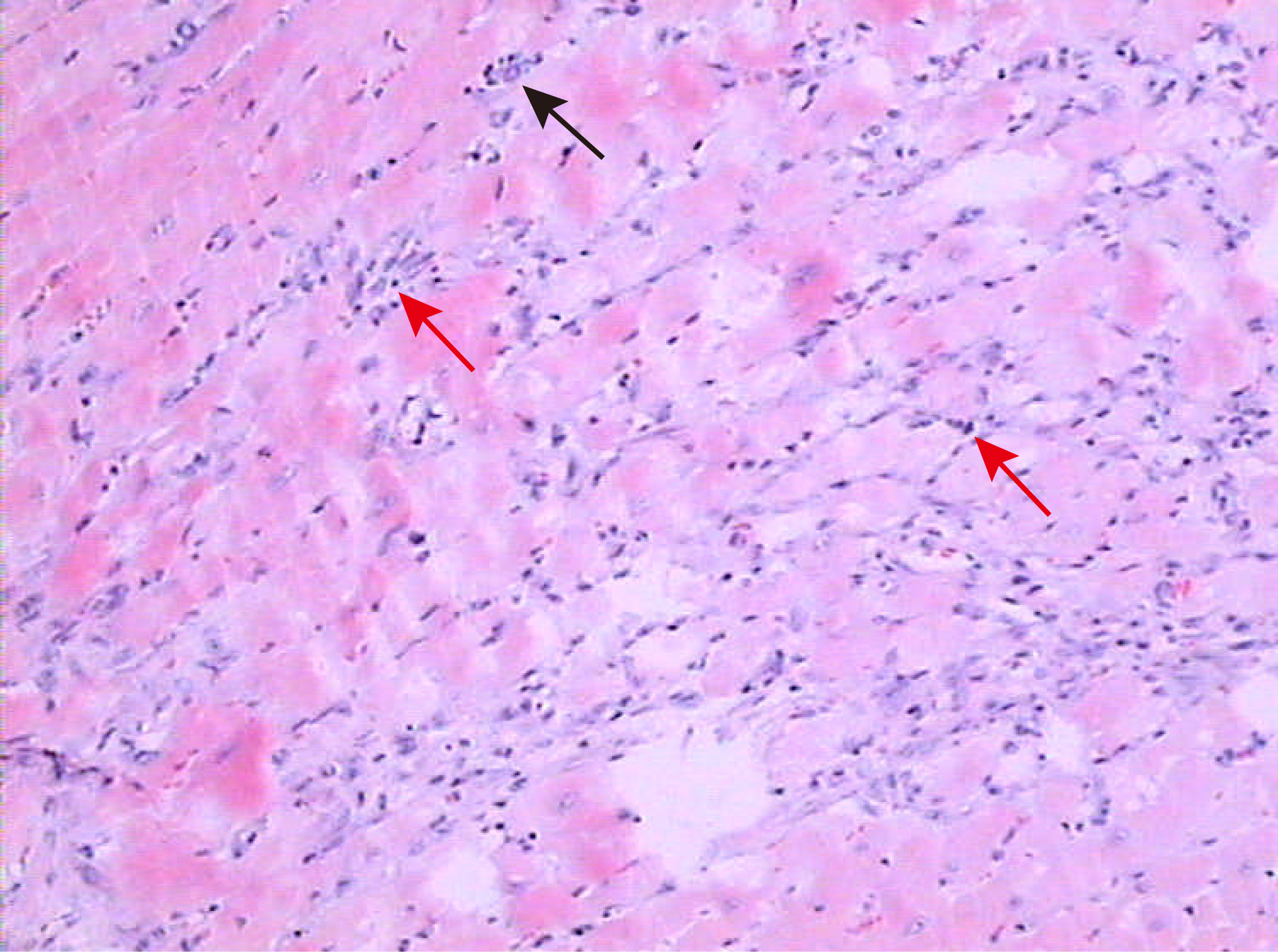

Supplement: Supplementary file 8 [file Data_Sheet_8.ZIP › Heart/D0.jpg]

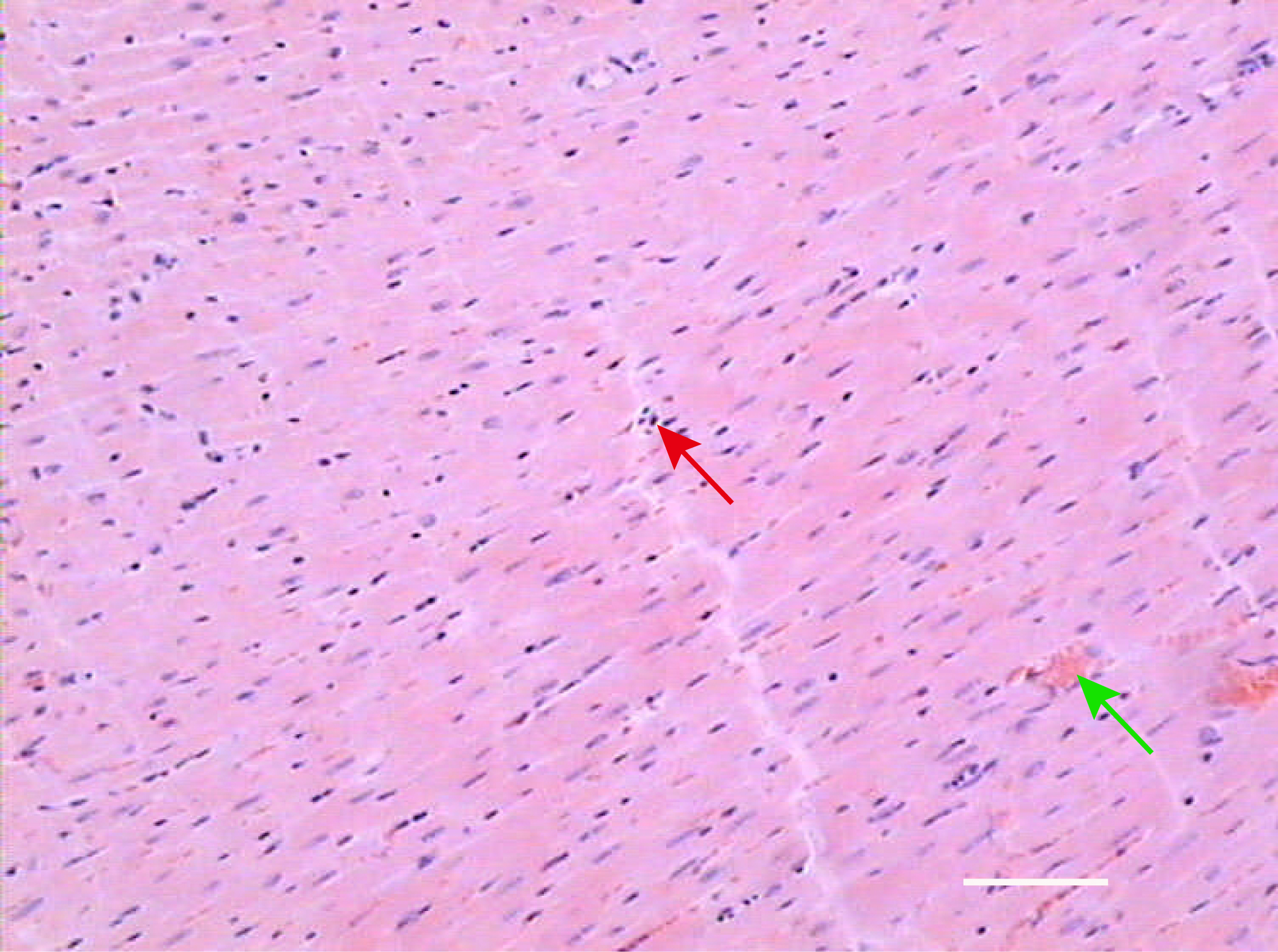

Supplement: Supplementary file 8 [file Data_Sheet_8.ZIP › Heart/D24.jpg]

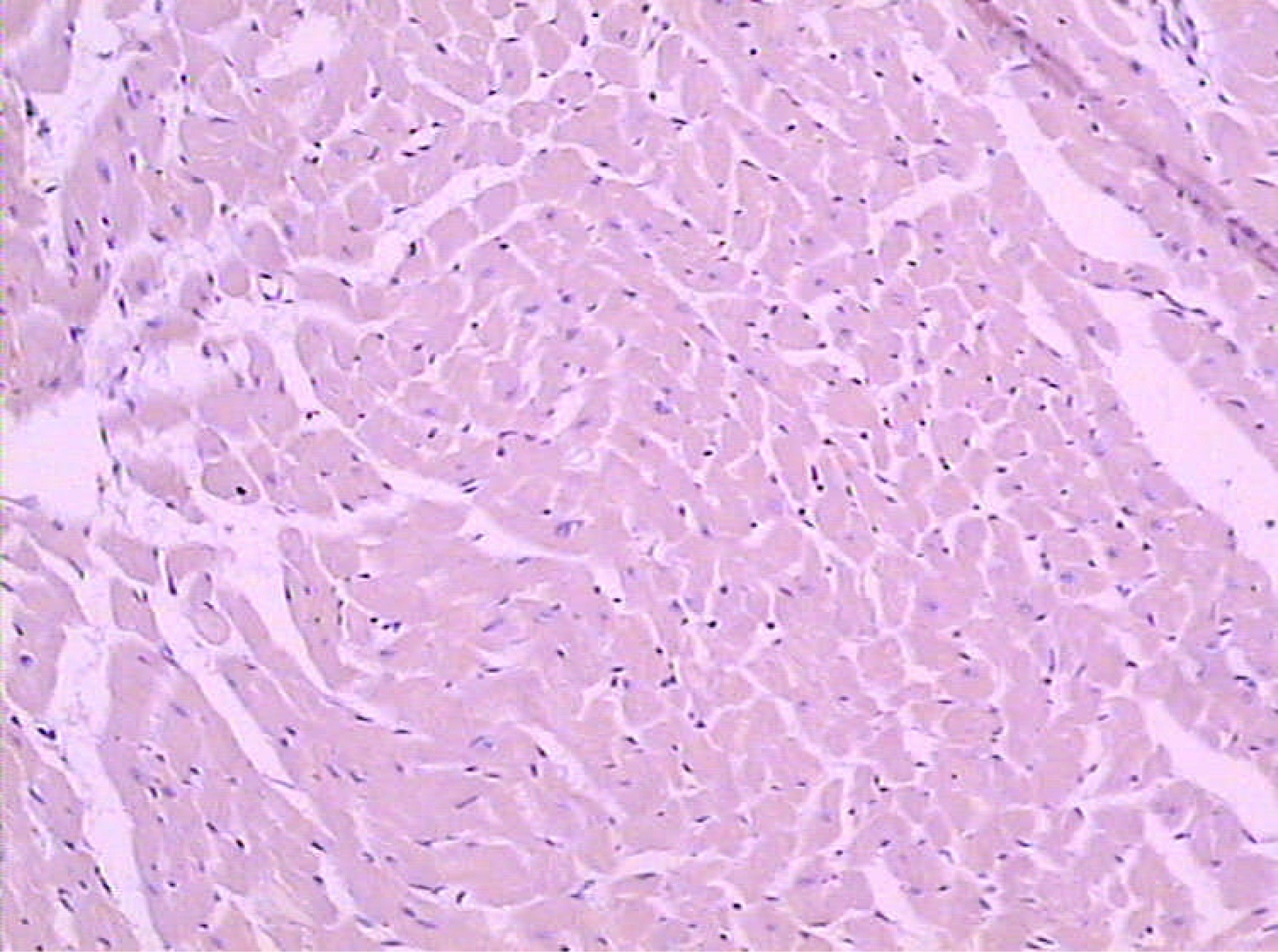

Supplement: Supplementary file 8 [file Data_Sheet_8.ZIP › Heart/S.jpg]

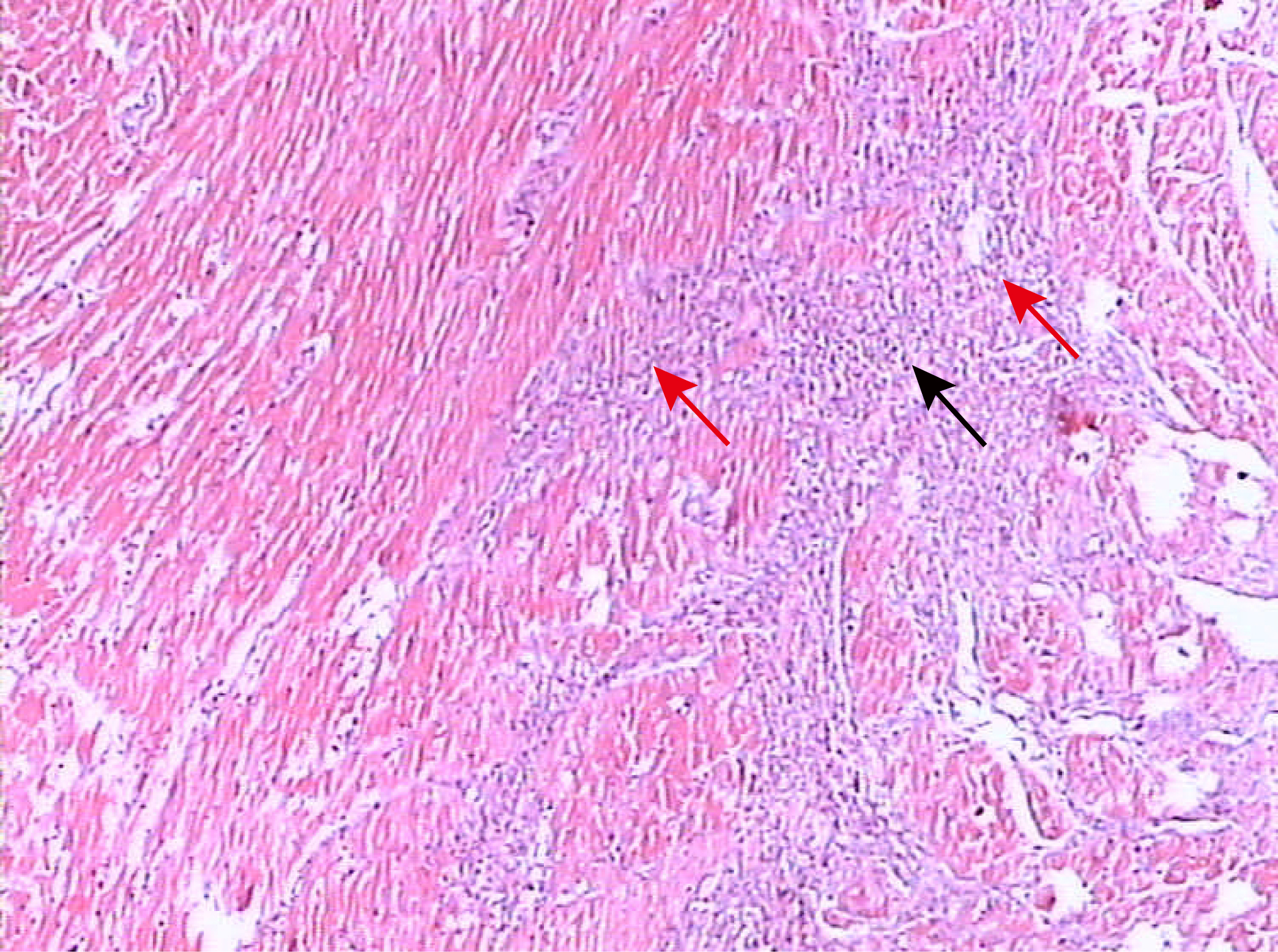

Supplement: Supplementary file 8 [file Data_Sheet_8.ZIP › Heart/U0.jpg]

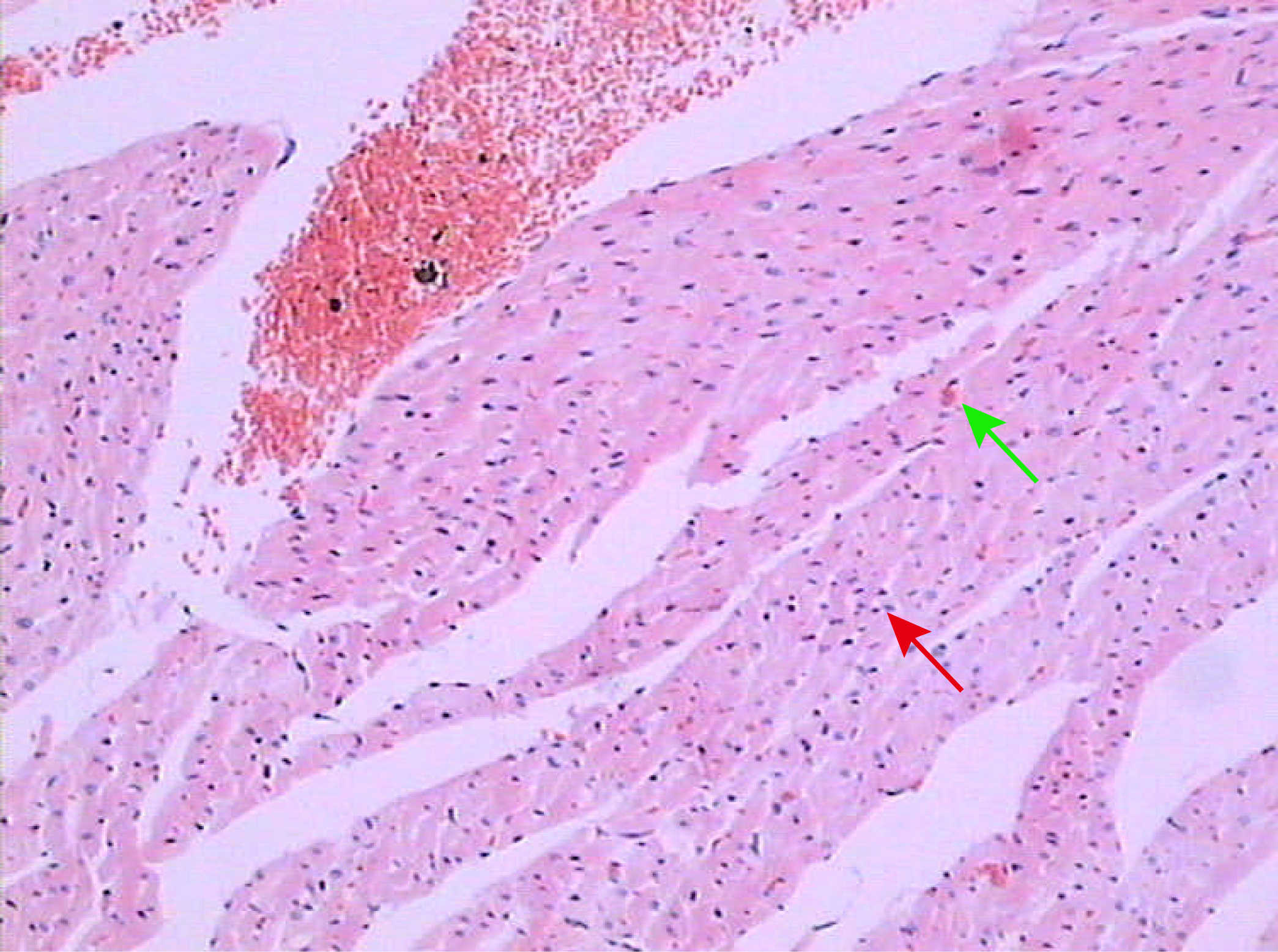

Supplement: Supplementary file 8 [file Data_Sheet_8.ZIP › Heart/U24.jpg]
